# Structural insights into the MLH1–FAN1 interaction reveal an uncharacterized binding interface on MLH1

**DOI:** 10.1038/s41467-026-74991-0

**Published:** 2026-07-06

**Authors:** Yichang Chen, Haiyun Hu, Xinci Shang, Keri M. Fishwick, Giada Greco, Qin Xiao, Yinhao Zhou, Qiuyan Huang, Tao Jiang, Xiaolei Huang, Guanghui Wang, Xuechu Zhen, Guoqiang Xu, Su Qin, Alessandro A. Sartori, Yanli Liu

**Affiliations:** 1 Jiangsu Key Laboratory of Drug Discovery and Translational Research for Brain Diseases, College of Pharmaceutical Sciences, Soochow University, Suzhou, Jiangsu China; 2 Institute of Molecular Cancer Research, University of Zurich, Zurich, Switzerland; 3 Life Science Research Center, Southern University of Science and Technology, Guangdong, China; 4 Jiangsu Province Engineering Research Center of Precision Diagnostics and Therapeutics Development, Soochow University, Suzhou, Jiangsu China; 5 Suzhou International Joint Laboratory for Diagnosis and Treatment of Brain Diseases, Suzhou, Jiangsu China

**Keywords:** DNA mismatch repair, X-ray crystallography, Huntington's disease

## Abstract

Huntington’s disease is driven by CAG repeat expansion in the mutant huntingtin gene. Nuclease FAN1 and mismatch repair protein MLH1 regulate repeat expansion through direct interaction, but the underlying structural basis remains unclear. Here, we show that the MLH1 C-terminal domain binds to FAN1-derived peptides containing either the MIP or MIM motif with comparable affinities. Crystal structures of this domain bound to each motif provide structural insights into human MLH1–FAN1 interaction, revealing a conserved mechanism for FAN1-MIP recognition and a previously unrecognized binding site on MLH1, termed the S3 site, for FAN1-MIM engagement. Co-immunoprecipitation assays confirmed that mutation of key MLH1 residues disrupts FAN1 binding in cells. These findings establish the molecular basis of MLH1–FAN1 recognition and provide a structural framework for understanding the regulation of CAG repeat expansion in Huntington’s disease.

## Abstract (web summary)

CAG repeat expansion in the huntingtin gene drives Huntington’s disease through interactions with DNA repair proteins. Here, the authors resolve structures of MLH1 bound to FAN1 motifs, defining distinct binding sites and the molecular basis of their interaction underlying repeat expansion regulation.

## Introduction

**Fig. 1:**
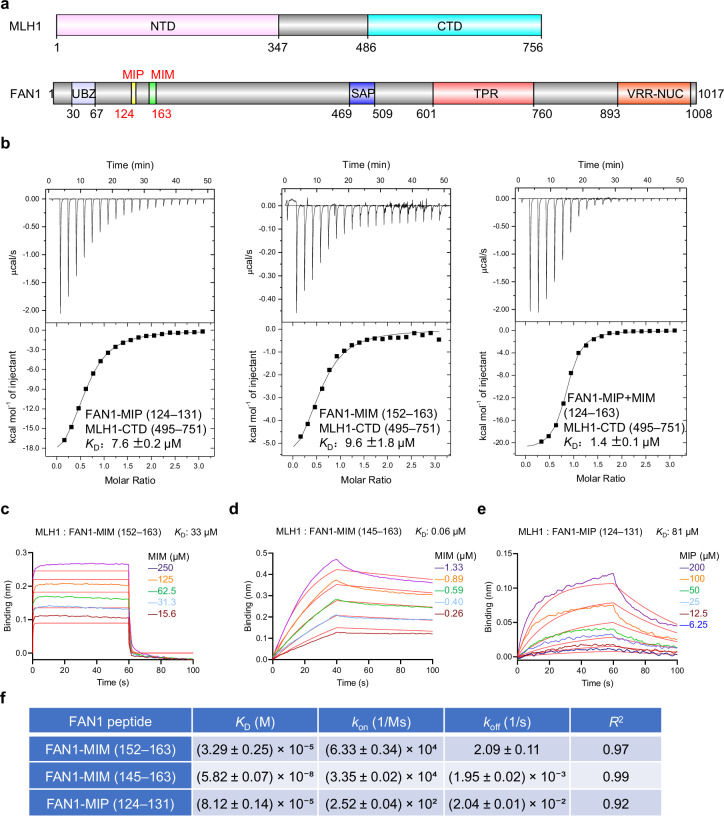
MLH1-CTD binds to FAN1-MIP and FAN1-MIM peptides with comparable affinities. **a** Schematic representation of the domain architecture of human MLH1 and FAN1. NTD, N-terminal domain; CTD, C-terminal domain; UBZ, ubiquitin-binding zinc finger; MIP, MLH1 interacting protein-box; MIM, MLH1 interacting motif; SAP, SAF-A/B, Acinus and PIAS domain; TPR, tetratricopeptide repeat domain; VRR-NUC, virus-type replication repair nuclease domain. **b** ITC binding curves for the titration of FAN1-MIP (residues 124–131), FAN1-MIM (residues 152–163), FAN1-MIP + MIM (residues 124–163) peptide to MLH1-CTD (residues 495–751), respectively. ITC data shown are representative of two independent experiments performed by iTC-200 microcalorimeter (MicroCal, Inc.). **c**–**e** BLI binding curves for biotinylated MLH1-CTD (residues 495–751) with FAN1-MIM short (residues 152–163, **c**), FAN1-MIM long (residues 145–163, **d**) or FAN1-MIP (residues 124–131, **e**), respectively. All the assays were run by using Octet-R2 instrument (Sartorius) at 30 °C with biotin-MLH1-CTD protein immobilized on a streptavidin biosensor and incubated with varying concentrations of FAN1 peptides. Data shown are representative of two independent experiments. **f** Binding data of different FAN1 peptides to biotin-MLH1-CTD determined by BLI. *K*_D_: dissociation constant. Source data are provided as a Source Data file.

MLH1 (MutL homolog 1) was first identified in 1994 by Bronner et al. during their study of hereditary non-polyposis colon cancer (HNPCC, also known as Lynch syndrome)^1^. As a key component of the DNA mismatch repair (MMR) system, a pathway conserved from bacteria to humans^2–4^, MLH1 plays a central role in genome maintenance. In bacteria, MutL forms a homodimer^5^, whereas in human (and yeast), there are four homologs of MutL, namely MLH1 (Mlh1), PMS2 (Pms1), PMS1 (Mlh2), and MLH3 (Mlh3), which form three heterodimeric MutL complexes: MutLα (MLH1–PMS2 in human, Mlh1–Pms1 in yeast), MutLβ (MLH1–PMS1 in human, Mlh1–Mlh2 in yeast) and MutLγ (MLH1–MLH3 in human, Mlh1–Mlh3 in yeast)^6–10^. The common member of these complexes, MLH1, is composed of 756 amino acid residues that are organized into an N-terminal domain (NTD) and a C-terminal domain (CTD), connected by a flexible linker (Fig. 1a). The NTD, referred to as the ATPase domain, contains the ATP-binding and ATP-hydrolyzing site^11–14^, while the CTD mediates heterodimerization and contributes to the endonuclease activity of Pms1 and Mlh3^15,16^. Both the ATPase activity of the NTD and endonuclease activity of the CTD of MutL complexes are essential for MMR. Within the CTD, the heterodimerization region is referred to as the S1 site, while a second region, the S2 site, is responsible for recruiting proteins that contain a conserved MIP-box motif (MLH1 interacting protein-box with a consensus sequence (R/K)SK(Y/F)F)^15–18^. Overall, MLH1 plays critical roles in post-replicative MMR and regulation of gene recombination^2–4^.

FAN1 (FANCD2/FANCI-associated nuclease 1) was first isolated from human brain cDNA libraries^19^. Subsequent studies indicate that FAN1 is a DNA nuclease with both endonuclease and 5′–3′ exonuclease activities. It is recruited to sites of DNA interstrand cross-links (ICLs) via the monoubiquitinated FANCD2–FANCI complex, hence its name^20–23^. FAN1 is composed of 1017 residues containing a ubiquitin-binding zinc finger (UBZ), an SAP (SAF-A/B, Acinus and PIAS) type DNA binding domain, a protein–protein interaction tetratricopeptide repeat (TPR) domain, and a virus type replication-repair nuclease (VRR-NUC) domain (Fig. 1a)^22,23^. Although FAN1 has originally been recognized as a nuclease implicated in the FA pathway of ICL repair, other studies demonstrate that FAN1 participates in ICL repair independently of the FA pathway and regulates the progression of stalled replication forks^24–27^. Moreover, FAN1 interacts physically with MLH1, suggesting that FAN1 may participate in MMR^22,23,28,29^.

Huntington’s disease (HD) is an autosomal dominantly inherited neurodegenerative disorder caused by the expansion of CAG trinucleotide repeats beyond 36 units in the mutant huntingtin (m*HTT*) gene^30,31^. Genome-wide and transcriptome-wide association studies (GWAS and TWAS) have consistently identified DNA repair genes as major modifiers of the onset and progression of HD^31–37^. Among these, MLH1 and FAN1 exhibit particularly strong associations. MLH1 promotes somatic CAG expansion and its protein levels influence the efficiency of this expansion^33,36^. In the *Htt*^*Q111*^ knock-in mouse models, both *Mlh1* and *Mlh3* genes have been shown to drive the expansion of CAG repeat sequences in the striatum^36^. Conversely, during the progression of HD, FAN1 expression exhibits a dose-dependent protective effect against CAG repeat expansion^32^. Overexpression of FAN1 in human cells decreases the CAG repeat expansion of the mutant *HTT* gene, while knockout of the *FAN1* gene in patient-derived induced pluripotent stem cells and differentiated neurons stimulates CAG repeat expansion^35^.

Recent studies demonstrate that MLH1 interacts with FAN1 by recognizing the MIP-box and MIM-motif (MLH1 interacting motif) in the N-terminus of FAN1 through its CTD to regulate CAG repeat expansion in HD (Fig. 1a)^38,39^. Elucidating the structural basis of the MLH1–FAN1 interaction is therefore essential for unraveling the molecular mechanisms regulating trinucleotide repeat expansion in HD.

In this study, we conducted quantitative binding assays and X-ray crystallography analyses of the human MLH1-CTD with the FAN1-MIP or FAN1-MIM motif. Our binding data confirm robust interactions between MLH1-CTD and both of these motifs, with significantly enhanced affinity observed for a FAN1 peptide comprising both motifs (residues 124–163). Furthermore, we determine the crystal structures of human MLH1-CTD in complex with each motif. These structures reveal a conserved recognition mode for FAN1-MIP across different species and identify a previously uncharacterized binding site, designated the S3 site, for engagement of FAN1-MIM. Co-immunoprecipitation assays further confirmed that mutation of key MLH1 residues disrupts FAN1 binding in a cellular context. Together, our findings uncover the molecular mechanism of the interaction between MLH1 and FAN1, and provide a structural foundation for understanding its role in HD pathogenesis.

## Results

### MLH1-CTD binds to FAN1-MIP/MIM peptides with comparable affinities

Previous study has shown that MLH1-CTD recognizes two key motifs in FAN1, the MIP-box (residues 124–131) and the MIM-motif (residues 152–163)^39^. To quantify these interactions, isothermal titration calorimetry (ITC) assays were conducted to determine the dissociation constant (*K*_D_) of the recombinant MLH1-CTD (residues 495–751) protein with synthetic FAN1-MIP, FAN1-MIM, and FAN1-MIP + MIM (residues 124–163, containing both motifs and the intervening linker) peptides. The ITC data revealed that FAN1-MIP and FAN1-MIM peptides bind to MLH1-CTD with similar affinities (*K*_D_ values of 7.6 μM and 9.6 μM, respectively, Fig. 1b). In contrast, the FAN1-MIP + MIM peptide exhibits a 5–7-fold stronger binding affinity (*K*_D_: 1.4 μM, Fig. 1b), consistent with previous findings^39^. Similar results were obtained using an extended MLH1-CTD fragment (residues 486–751) in ITC assays with the same peptides and the recombinant FAN1 (residues 118–177) protein (Supplementary Fig. 1), supporting the idea that MLH1-CTD can simultaneously engage both motifs. Furthermore, co-expression of MLH1-CTD (residues 495–751) and FAN1 (residues 118–177) allowed successful reconstitution of the binary complex, further confirming their interaction (Supplementary Fig. 2).

A prior study reported that the N-terminal region of MSH3, a component of the MutSβ complex, contains a MIP-box motif responsible for the MSH3–MLH1 interaction^40^. To evaluate the binding affinity of MSH3-MIP with MLH1-CTD and its potential influence on FAN1–MLH1 interaction, we performed competitive ITC assays with MSH3-MIP (residues 23–30) and FAN1-MIP peptides. The ITC data showed that MSH3-MIP binds to MLH1-CTD with much weaker binding affinity compared to FAN1-MIP and the interaction of MSH3–MLH1 is substantially weakened in the presence of the FAN1-MIP peptide (Supplementary Fig. 3a). Conversely, the addition of MSH3-MIP shows a negligible effect on the interaction of FAN1–MLH1 (Supplementary Fig. 3b). Taken together, these findings demonstrate that FAN1 competes with MSH3 for the binding site on MLH1 and may thereby block the interaction of MSH3–MLH1, in line with earlier reports^38^.

We further analyzed the binding of FAN1-MIM peptides of different lengths to MLH1-CTD. Although ITC detected binding for the shorter FAN1-MIM peptide (residues 152–163), it did not yield a measurable signal for the longer peptide (residues 145–163) under the experimental conditions used. We therefore employed biolayer interferometry (BLI) with biotinylated MLH1-CTD to characterize these interactions kinetically. BLI revealed that the shorter peptide (residues 152–163) binds with rapid association and dissociation kinetics. In contrast, the longer peptide (residues 145–163) exhibits a comparable association rate (*k*_on_, (3.35 ± 0.02) × 10⁴ M⁻¹s⁻¹ *vs*. (6.33 ± 0.34) × 10⁴ M⁻¹s⁻¹ for the shorter peptide) but a dramatically slower dissociation rate (*k*_off_ = (1.95 ± 0.02) × 10⁻³ s⁻¹), nearly three orders of magnitude slower than that of the shorter peptide (*k*_off_ = 2.09 ± 0.11 s⁻¹) (Fig. 1c, d, f). The substantially slow dissociation of the longer peptide likely prevented the system from reaching binding equilibrium within the ITC experimental timeframe, explaining the lack of a detectable signal. BLI is particularly suited for characterizing interactions with very slow dissociation rates. Consistent with its slow dissociation, the longer peptide also shows a markedly higher binding affinity than the shorter one (*K*_D_: 0.06 μM *vs*. 33 μM, Fig. 1c, d, f), suggesting that the FAN1_145–151 segment preceding the FAN1-MIM sequence (residues 152–163) significantly stabilizes the interaction with MLH1-CTD. Notably, the BLI-derived affinity of the shorter FAN1-MIM peptide (*K*_D_ = 33 µM, Fig. 1c) was weaker than that determined by ITC (9.6 µM, Fig. 1b). To examine whether this was method-dependent, we also measured the affinity of FAN1-MIP (residues 124–131) by BLI, which similarly yielded a weaker value (81 µM, Fig. 1e) compared to ITC (7.6 µM, Fig. 1b). Both ITC and BLI independently confirmed that the FAN1-MIP and FAN1-MIM motifs bind to MLH1-CTD. Although the absolute affinity values varied between techniques, a common occurrence due to their distinct measurement principles^41^, each method provided self-consistent relative comparisons. For instance, both techniques agreed that FAN1-MIP and the core FAN1-MIM motif bind with comparable micromolar-range affinity, and BLI further established that the extended MIM peptide binds with much higher affinity. Collectively, these data demonstrate that MLH1-CTD engages both FAN1 motifs productively and that FAN1 can directly interfere with the MLH1–MSH3 interaction.

### FAN1-MIP (residues 124–131) binds to the S2 site of MLH1-CTD

**Fig. 2:**
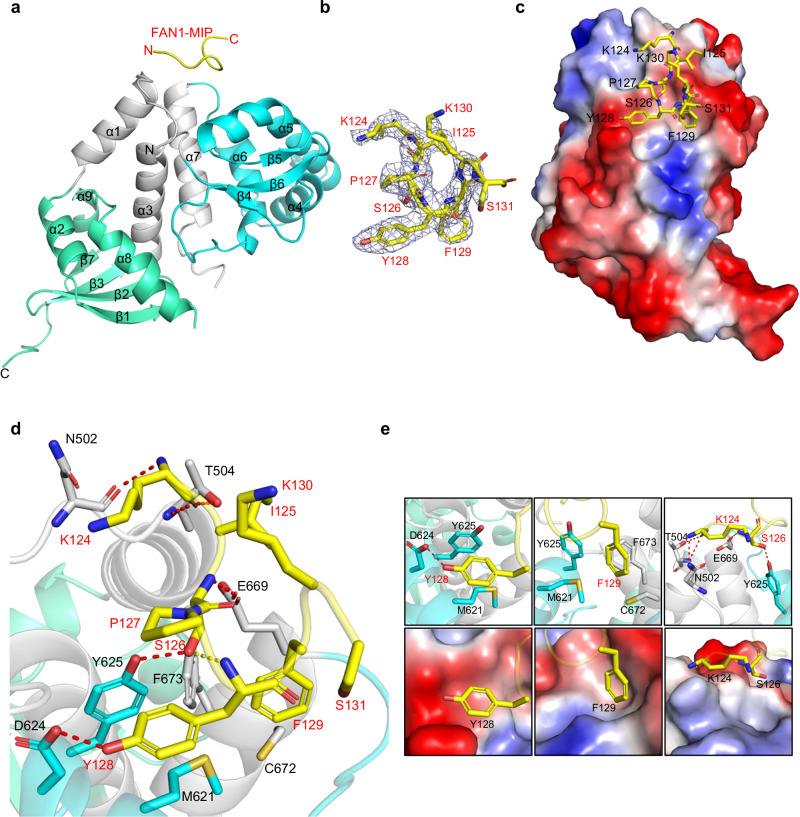
Crystal structure of MLH1-CTD (residues 495–751) in complex with FAN1-MIP (residues 124–131). **a** Overall structure of MLH1-CTD in complex with FAN1-MIP peptide. The protein and peptide are shown in cartoon representation with MLH1-CTD colored in cyan, green-cyan, gray for the regulatory subdomain, dimerization subdomain and inter-subdomain linker, and FAN1-MIP peptide colored in yellow, respectively. **b** ${2F}_{o}-{F}_{c}$ electron density map of FAN1-MIP peptide contoured at 1*σ* level. **c** Electrostatic potential surface representation of the complex of MLH1-CTD and FAN1-MIP with FAN1-MIP peptide shown in stick mode. **d** Detailed interactions of the FAN1-MIP peptide with MLH1-CTD. The FAN1-MIP peptide and peptide-interacting MLH1 residues are shown in stick mode, the intermolecular and intramolecular hydrogen bonds are indicated as red and yellow dashed lines, respectively. **e** Key residue interactions in the MLH1-CTD–FAN1-MIP interface. FAN1 and MLH1 amino acid residues are labeled in red and black, respectively. Structure figures were generated by using PyMOL. Electrostatic potential surface representations were calculated with PyMOL’s built-in protein contact potential function with negatively and positively charged regions shown in red and blue, respectively^64^.

To illustrate the molecular mechanism of the interaction between MLH1-CTD and FAN1-MIP peptide, we attempted to crystallize MLH1-CTD fragments of varying lengths in complex with the FAN1-MIP peptide and solved the complex crystal structure of MLH1-CTD (residues 495–751) and the FAN1-MIP (residues 124–131) peptide (Fig. 2, Supplementary Fig. 4a, b and Supplementary Table 1). The overall structure of human MLH1-CTD is similar to that of previously reported MutL homologs, comprising an internal dimerization subdomain and an external regulatory subdomain^15,42–44^. The dimerization subdomain consists of a β-sheet formed by four antiparallel β-strands (β1, β2, β3, and β7) and three α-helices (α2, α8, and α9). The external regulatory subdomain contains a β-sheet composed of three antiparallel β-strands (β4–β6) and three α-helices (α4–α6), with helices α1, α3 and α7 connecting these two subdomains (Fig. 2a).

In the complex, FAN1-MIP interacts with MLH1 helices α1, α5, and α7, namely the S2 site, similar to the previously reported complex structure of *S. cerevisiae* Mlh1-CTD and MIP peptides^15^. The residues 124 to 131 of FAN1-MIP could be traced by the electron density and encompassed by the S2 site (Fig. 2b–d). The FAN1-MIP peptide adopts an ST-turn conformation^45^, where the side chain of S126 (position i) forms an intramolecular hydrogen bond with the main-chain amide group of Y128 at position i + 2 (Fig. 2d). The interaction between FAN1-MIP and MLH1 is primarily mediated by hydrophobic stacking and hydrogen bonds. The side chain of FAN1-Y128 engages in hydrophobic stacking with MLH1-M621and MLH1-Y625, while the side chain of FAN1-F129 inserts into a hydrophobic pocket formed by MLH1-M621, MLH1-Y625, MLH1-C672 and MLH1-F673 (Fig. 2c–e). Additionally, several intermolecular hydrogen bonds between side chains of FAN1-Y128 and MLH1-D624, FAN1-S126 and MLH1-Y625, main chains of FAN1-K124 and MLH1-N502, MLH1-T504, main chain of FAN1-S126 and side chain of MLH1-E669, main chain of FAN1-K124 and side chain of MLH1-T504, further stabilize the complex (Fig. 2d, e). In summary, the complex structure of MLH1-CTD and FAN1-MIP reveals that the human MLH1-CTD recognizes the MIP motif by its S2 site, mirroring the binding mechanism previously described for yeast Mlh1-CTD.

### Mutations of the essential interacting residues disrupt the binding

**Fig. 3:**
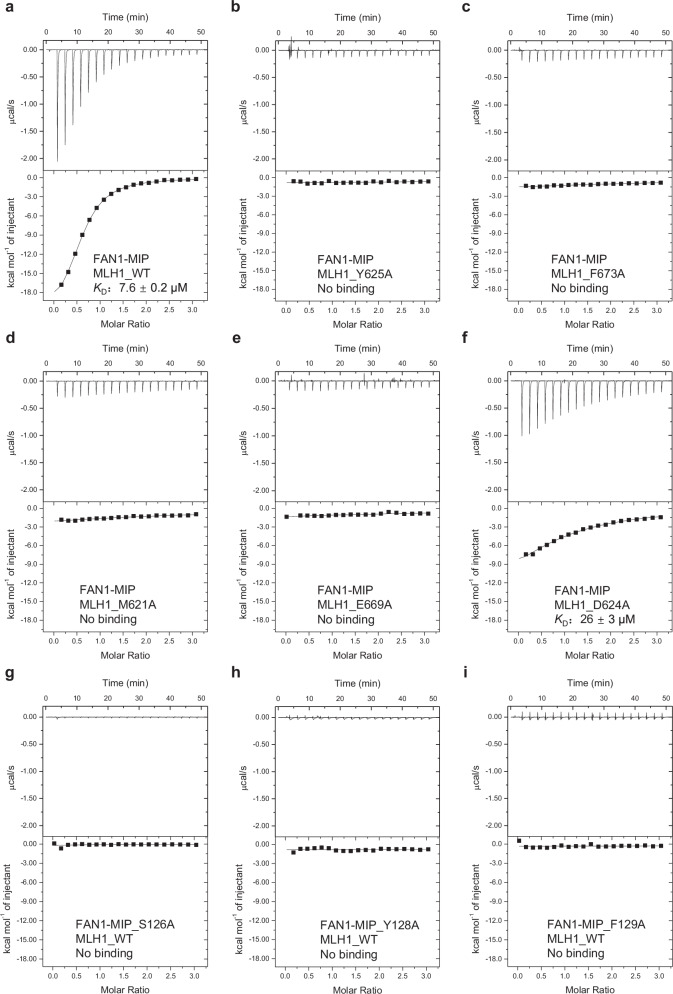
The interaction between MLH1-CTD and FAN1-MIP is mediated by the S2 site of MLH1-CTD and the SxYF motif of FAN1 MIP-box. **a**–**f** ITC binding curves for the wild-type FAN1-MIP peptide titrated to MLH1-CTD_WT (wild-type protein shown as a control, **a**), MLH1-CTD_Y625A (**b**), MLH1-CTD_F673A (**c**), MLH1-CTD_M621A (**d**), MLH1-CTD_E669A (**e**), and MLH1-CTD_D624A (**f**) mutant proteins, respectively. **g**–**i** ITC binding curves for the FAN1-MIP_S126A (**g**), FAN1-MIP_Y128A (**h**), and FAN1-MIP_F129A (**i**) mutant peptides titrated to the wild-type MLH1-CTD protein, respectively. *K*_D_: dissociation constant (μM); ITC data shown are representative of two independent experiments performed by iTC-200 microcalorimeter (MicroCal, Inc.).

To validate the findings from our complex crystal structure analyses, we introduced specific point mutations into MLH1-CTD and performed binding assays. Single point mutations of Y625A, F673A, and M621A in MLH1-CTD completely abolish the interaction with FAN1-MIP (Fig. 3a–d), highlighting the essential role of hydrophobic and stacking interactions between Y128 and F129 of FAN1 and these key MLH1 residues. Similarly, the E669A mutation in MLH1-CTD also fully disrupts the interaction (Fig. 3e), confirming the importance of the hydrogen bond between MLH1-E669 and FAN1-S126 (Fig. 2d, e). Furthermore, the mutation of D624 (forming a side-chain hydrogen bond with FAN1-Y128, Fig. 2d, e) reduces the binding affinity to 26 μM, weakening the binding by 3.4 folds relative to the wild type (Fig. 3f).

To further confirm the role of the SxYF motif in FAN1-MIP for MLH1-CTD binding, single-point mutations were introduced into the FAN1-MIP peptide and determined the binding affinity with wild-type MLH1-CTD by ITC. Mutations of S126A, Y128A, and F129A result in a complete loss of interaction with MLH1-CTD (Fig. 3g–i). These findings align with both our complex structure (Fig. 2) and prior study indicating the critical role of these residues in the recognition of the MIP-box by yeast Mlh1-CTD^15^. Additionally, our structure and mutation analyses also suggested that phosphorylation of FAN1-S126 would disrupt the hydrogen bond with MLH1-Y625 and introduce steric hindrance to destroy the interaction, consistent with the recent study^39^. Overall, these binding assays confirm the specific recognition of the SxYF motif in FAN1-MIP by the MLH1-CTD S2 site.

### FAN1-MIM (residues 145–163) binds to an uncharacterized site on MLH1

**Fig. 4:**
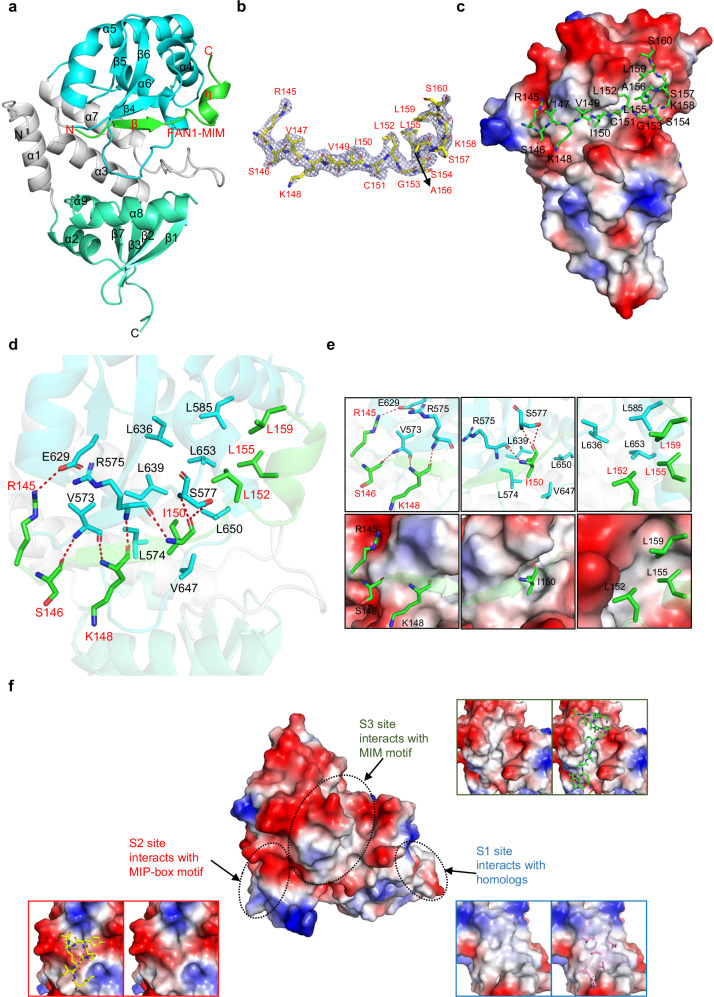
Crystal structure of MLH1-CTD (residues 495–751) in complex with FAN1-MIM (residues 145–163). **a** Overall structure of MLH1-CTD in complex with FAN1-MIM peptide. The protein and peptide are shown in cartoon representation with MLH1-CTD colored in cyan, green-cyan, gray for the regulatory subdomain, dimerization subdomain and inter-subdomain linker, and FAN1-MIM peptide colored in green, respectively. **b** ${2F}_{o}-{F}_{c}$ electron density map of FAN1-MIM peptide contoured at 1*σ* level. **c** Electrostatic potential surface representation of the complex of MLH1-CTD and FAN1-MIM with FAN1-MIM peptide shown in stick mode. **d** Detailed interactions of the FAN1-MIM peptide with MLH1-CTD. The FAN1-MIM peptide and peptide-interacting MLH1 residues are shown in stick mode, the intermolecular hydrogen bonds are shown as red dashed lines. **e** Key residue interactions in the MLH1-CTD–FAN1-MIM interface. FAN1 and MLH1 amino acid residues are labeled in red and black, respectively. **f** Electrostatic potential surface representation of MLH1-CTD, with the S1, S2, and S3 sites indicated by black dashed circles, and enlarged views shown for both the ligand-free and ligand-bound states. The ligand-bound form of the S1 site is modeled using the ScMlh3 fragment (colored in pink) from the heterodimer structure (PDB: 6RMN). Structure figures were generated by using PyMOL. Electrostatic potential surface representations were calculated with PyMOL’s built-in protein contact potential function with negatively and positively charged regions shown in red and blue, respectively^64^. Abbreviations: Sc, *Saccharomyces cerevisiae* (Baker’s yeast).

To elucidate the molecular mechanism underlying the interaction between MLH1-CTD and the FAN1-MIM peptide, we attempted to co-crystallize MLH1-CTD (residues 495–751) with the FAN1-MIM peptides of varying lengths and solved the crystal structure of the MLH1-CTD and FAN1-MIM (residues 145–163) peptide complex (Fig. 4, Supplementary Fig. 4c, d and Supplementary Table 1). In the complex, MLH1-CTD adopts a fold similar to that observed in the MLH1-CTD–FAN1-MIP complex, while the FAN1-MIM peptide could be distinctly traced from the residues 145 to 160 according to the electron density, which form a β-strand (β, residues V147–I150) followed by a 3_10_ helix (η, residues S154–K158) (Fig. 4a, b). To our surprise, the FAN1-MIM peptide binds neither to the S2 site in the regulatory subdomain nor to the S1 site in the dimerization subdomain^15–18^, but to a previously unrecognized site on MLH1-CTD, which we designate the S3 site (Fig. 4a, c). The FAN1-MIM β-strand aligns parallelly to MLH1-β4 and inserts into the cleft between the regulatory and dimerization subdomains, while FAN1-MIM η-helix projects into a helical bundle formed by MLH1-α4 and MLH1-α6 within the regulatory subdomain (Fig. 4a).

FAN1-MIM interacts with MLH1-CTD through extensive intermolecular hydrogen bonds between FAN1-MIM β-strand and MLH1-β4, along with prominent hydrophobic interactions involving FAN1-MIM η-helix and MLH1-α4 and MLH1-α6. Specifically, hydrogen bonds form between the main chains of FAN1 residues S146, K148, and I150, as well as the side chain of FAN1-R145, and the main chains of MLH1 residues V573, R575, and S577, together with the side chains of MLH1-S577 and E629 (Fig. 4d, e). Notably, FAN1-R145 forms a hydrogen bond with MLH1-E629 and fits optimally with the negatively charged surface of MLH1. The R145H mutation, which replaces a positively charged arginine with a histidine (carrying less positive charge at physiological conditions), is predicted to destabilize this interaction by reducing electrostatic complementarity, a finding supported by BLI assays (Supplementary Fig. 5). This provides a direct structural rationale for the observed correlation between the R145H variant and an earlier, more severe HD phenotype^46^. Concurrently, key hydrophobic contacts stabilize the complex. The side chain of FAN1-I150 inserts into a hydrophobic pocket on MLH1 formed by residues L574, L639, V647, and L650. Further hydrophobic stacking is observed between the C-terminal residues of FAN1-MIM (L152, L155, L159) and MLH1 residues L585, L636, and L653 (Fig. 4d, e). Collectively, the upstream segment (residues 145–151) of FAN1-MIM (residues 145–163) peptide critically stabilizes the complex through a network of extensive intermolecular hydrogen bonds and prominent hydrophobic interactions. This is fully consistent with our binding assays, which reveals that longer FAN1-MIM (residues 145–163) peptide shows a markedly higher binding affinity than the shorter one (*K*_D_: 0.06 μM *vs*. 33 μM, Fig. 1c, d, f). In summary, the complex structure reveals that the FAN1-MIM peptide binds to a previously uncharacterized region on MLH1-CTD, designated the S3 site. This interaction involves both the core MIM motif (residues 152–163) and its preceding segment (residues 145–151), and is stabilized by a complementary combination of specific hydrogen bonds and hydrophobic interactions.

Combining these structural studies, a summary of MLH1-CTD binding site on MLH1-CTD was generated (Fig. 4f). MLH1-CTD forms heterodimers via hydrophobic packing mediated by the β-sheet (β1, β2, β3, and β7) within its dimerization subdomain (Fig. 4f)^15,16^. Meanwhile, it engages the MIP-box through hydrophobic stacking and hydrogen bonds involving helices α1, α5, and α7 of the regulatory subdomain (Figs. 2 and 4f). Furthermore, the MIM motif is accommodated by the β4 strand and the cleft between the regulatory and dimerization subdomains, as well as through interactions with the helical bundle formed by MLH1-α4 and MLH1-α6 within the regulatory subdomain, all stabilized by extensive intermolecular hydrogen bonds and prominent hydrophobic contacts (Fig. 4). Collectively, MLH1-CTD utilizes distinct structural regions to interact with different ligands, thereby functioning as a versatile protein interaction hub.

### FAN1-MIM motif and its upstream segment essential for MLH1 binding

**Fig. 5:**
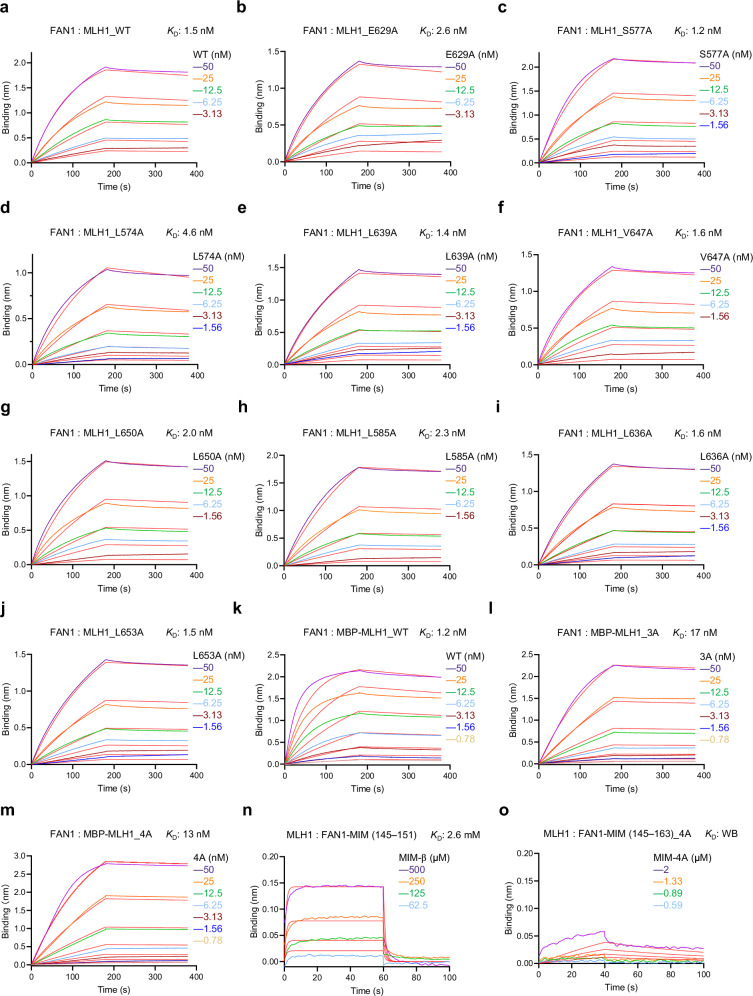
The interaction between MLH1-CTD and FAN1-MIM involves a previously uncharacterized site on MLH1-CTD and two segments of FAN1-MIM. **a**–**m** BLI binding curves for C-terminally biotinylated FAN1-MIM (residues 145–163) peptide with MLH1-CTD_WT (**a**), MLH1-CTD_E629A (**b**), MLH1-CTD_S577A (**c**), MLH1-CTD_L574A (**d**), MLH1-CTD_L639A (**e**), MLH1-CTD_V647A (**f**), MLH1-CTD_L650A (**g**), MLH1-CTD_L585A (**h**), MLH1-CTD_L636A (**i**), MLH1-CTD_L653A (**j**), MBP-MLH1-CTD_WT (**k**), MBP-MLH1-CTD_3A (**l**), MBP-MLH1-CTD_4A (**m**), respectively. **n**, **o** BLI binding curves for N-terminally biotinylated MLH1-CTD (residues 495–751) with FAN1-MIM (residues 145–151, **n**), and FAN1-MIM (residues 145–163)_4A, **o**), respectively. All the assays were run by using Octet-R2 instrument (Sartorius) at 30 °C with FAN1-MIM (residues 145–163)-biotin peptide immobilized onto a streptavidin biosensor and incubated with varying concentrations of MLH1-CTD proteins for **a**–**m** or with biotin-MLH1-CTD protein immobilized onto a streptavidin biosensor and incubated with varying concentrations of FAN1 peptides for **n**,** o**. MBP-MLH1_WT: MBP-tag fused MLH1-CTD; MBP-MLH1_3A: MBP-tag fused MLH1-CTD with L585A/L636A/L653A three-point mutations; MBP-MLH1_4A: MBP-tag fused MLH1-CTD with L574A/L639A/V647A/L650A four-point mutations; FAN1-MIM (145–163)_4A: FAN1-MIM (residues 145–163) peptide with I150A/L152A/L155A/L159A four-point mutations; WB: weak binding. Data shown are representative of two independent experiments. Source data are provided as a Source Data file.

To verify the structural insights from the MLH1–FAN1-MIM complex, we conducted binding assays using BLI with C-terminally biotin-labeled FAN1-MIM (residues 145–163) peptide and the wild-type or mutant MLH1-CTD proteins. BLI data revealed that single-point mutations of MLH1 residues involved in side-chain interactions do not significantly reduce binding affinity relative to wild-type MLH1 (Fig. 5a–j and Table 1), suggesting that individual mutations of MLH1 are insufficient to effectively disrupt the interaction between FAN1-MIM and MLH1-CTD.

**Table 1:** Binding data of wild-type and mutant MLH1-CTD proteins to FAN1-MIM (residues 145–163)-biotin determined by BLI

Role in FAN1 interaction	MLH1-CTD protein	*K*_D_ (M)	*k*_on_ (1/Ms)	*k*_off_ (1/s)	*R*²
	MLH1_WT	(1.50 ± 0.04) × 10⁻⁹	(2.08 ± 0.01) × 10⁵	(3.11 ± 0.09) × 10⁻⁴	0.99
H-bond with FAN1_R145	MLH1_E629A	(2.61 ± 0.11) × 10⁻⁹	(1.56 ± 0.01) × 10⁵	(4.07 ± 0.17) × 10⁻⁴	0.96
H-bond with FAN1_I150	MLH1_S577A	(1.21 ± 0.02) × 10⁻⁹	(1.62 ± 0.01) × 10⁵	(1.97 ± 0.03) × 10⁻⁴	0.99
Hydrophobic cage for FAN1-I150	MLH1_L574A	(4.60 ± 0.05) × 10⁻⁹	(1.12 ± 0.01) × 10⁵	(5.16 ± 0.05) × 10⁻⁴	0.99
	MLH1_L639A	(1.41 ± 0.07) × 10⁻⁹	(1.39 ± 0.01) × 10⁵	(1.97 ± 0.09) × 10⁻⁴	0.97
	MLH1_V647A	(1.56 ± 0.04) × 10⁻⁹	(1.60 ± 0.01) × 10⁵	(2.49 ± 0.07) × 10⁻⁴	0.97
	MLH1_L650A	(2.04 ± 0.03) × 10⁻⁹	(1.27 ± 0.01) × 10⁵	(2.58 ± 0.04) × 10⁻⁴	0.98
Hydrophobic interaction with C terminal of FAN1-MIM motif	MLH1_L585A	(2.33 ± 0.04) × 10⁻⁹	(9.00 ± 0.05) × 10⁴	(2.10 ± 0.03) × 10⁻⁴	0.99
	MLH1_L636A	(1.62 ± 0.03) × 10⁻⁹	(1.08 ± 0.01) × 10⁵	(1.75 ± 0.03) × 10⁻⁴	0.99
	MLH1_L653A	(1.46 ± 0.03) × 10⁻⁹	(1.18 ± 0.01) × 10⁵	(1.71 ± 0.04) × 10⁻⁴	0.98
	MBP-MLH1_WT	(1.22 ± 0.01) × 10⁻⁹	(3.48 ± 0.01) × 10⁵	(4.23 ± 0.03) × 10⁻⁴	0.99
Key residues for MIM C-ter	MBP-MLH1_3A	(1.69 ± 0.03) × 10⁻⁸	(8.47 ± 0.03) × 10³	(1.43 ± 0.02) × 10⁻⁴	0.99
Key residues for FAN1-I150	MBP-MLH1_4A	(1.30 ± 0.02) × 10⁻⁸	(8.93 ± 0.02) × 10³	(1.16 ± 0.02) × 10⁻⁴	0.99

MBP-MLH1_WT: MBP-tag fused MLH1-CTD; MBP-MLH1_3A: MBP-tag fused MLH1-CTD with L585A/L636A/L653A three-point mutations, targeting the stacking interface for FAN1-MIM C-terminus; MBP-MLH1_4A: MBP-tag fused MLH1-CTD with L574A/L639A/V647A/L650A four-point mutations, targeting hydrophobic cage for FAN1-I150.

We therefore generated two multi-residue MLH1 mutants, L585A/L636A/L653A (referred to as MLH1_3A) and L574A/L639A/V647A/L650A (referred to as MLH1_4A), designed to disrupt the two key hydrophobic interfaces between FAN1-MIM and MLH1-CTD, namely the FAN1-I150 hydrophobic cage (targeted by MLH1_4A) and the hydrophobic patch accommodating the C-terminus of FAN1-MIM (targeted by MLH1_3A) (Supplementary Fig. 6a, b). When expressed using the pET28-MHL vector (employed for the wild-type MLH1-CTD), both mutant proteins formed inclusion bodies and could not be purified in an active form through refolding. Structural analyses suggested that the mutated residues contribute to the hydrophobic core, with L636 in the MLH1_3A mutant and L639 in the MLH1_4A mutant being particularly critical (Supplementary Fig. 6a, b), consistent with the low yield observed for individual mutations at these positions. Consequently, mutating multiple residues, including these core ones as in MLH1_3A and MLH1_4A, is likely to destabilize the MLH1-CTD fold. This instability provides a plausible explanation for the formation of inclusion bodies upon overexpression in *E. coli*. To address the solubility issue, we subsequently constructed fusion protein expression vectors containing an N-terminal MBP (maltose-binding protein, a well-established solubility enhancer) tag and obtained the soluble MBP-MLH1 fusion proteins (Supplementary Fig. 6c). Circular dichroism (CD) spectroscopy confirmed that the mutants retained a wild-type-like folded structure (Supplementary Fig. 6d). Subsequent BLI assays using these MBP-fused proteins demonstrated that both the MLH1_3A and MLH1_4 A mutations reduce the binding affinity for FAN1-MIM by approximately 10-fold compared to wild-type MLH1 (Fig. 5k–m and Table 1).

We further evaluated the binding abilities of truncated and mutant FAN1-MIM (residues 145–163) peptides to biotin-labeled MLH1-CTD by BLI. The data showed that FAN1-MIM_β (residues 145–151) only weakly binds to MLH1-CTD, while the quadruple mutant FAN1-MIM-4A (I150A/L152A/L155A/L159A), in which the key hydrophobic residues were substituted, almost abrogates the interaction (Fig. 5n, o and Table 2). These results underscore the essential contribution of hydrophobic interactions to complex stability. Moreover, the full FAN1-MIM_β + η (residues 145–163) exhibits significantly stronger binding than the minimal MIM motif (residues 152–163, FAN1-MIM_η) to MLH1-CTD (Fig. 1c, d and Table 2), indicating that the residues 145–151 of FAN1 significantly enhance binding within the extended FAN1-MIM (residues 145–163) context, consistent with our structural analyses (Fig. 4). Overall, these quantified binding data demonstrate that the MLH1-CTD–FAN1-MIM interaction is stabilized by both intermolecular hydrogen bonds and extensive hydrophobic contacts, with both the core MIM motif and its N-terminal flanking segment playing indispensable roles.

**Table 2:** Binding data of different FAN1 peptides to biotin-MLH1-CTD (residues 495–751) determined by BLI

FAN1 peptide	*K*_D_ (M)	*k*_on_ (1/Ms)	*k*_off_ (1/s)	*R*²
FAN1-MIM (145–163, MIM-β + η)	(5.82 ± 0.07) × 10⁻⁸	(3.35 ± 0.02) × 10⁴	(1.95 ± 0.02) × 10⁻³	0.99
FAN1-MIM (145–151, MIM-β)	(2.64 ± 0.19) × 10⁻³	(3.26 ± 0.22) × 10²	(8.62 ± 0.24) × 10⁻¹	0.97
FAN1-MIM (152–163, MIM-η)	(3.29 ± 0.25) × 10⁻⁵	(6.33 ± 0.34) × 10⁴	2.09 ± 0.11	0.97
FAN1-MIM (145–163)_4A	Weak binding	--	--	--
FAN1-MIM (145–163)_R145H	Weak binding	--	--	--
FAN1-MIP (124–131)	(8.12 ± 0.14) × 10⁻⁵	(2.52 ± 0.04) × 10²	(2.04 ± 0.01) × 10⁻²	0.92

FAN1-MIM (145–163)_4A: FAN1-MIM (residues 145–163) peptide with I150A/L152A/L155A/L159A four-point mutations.

### Key residue mutations impair cellular MLH1–FAN1 interaction

**Fig. 6:**
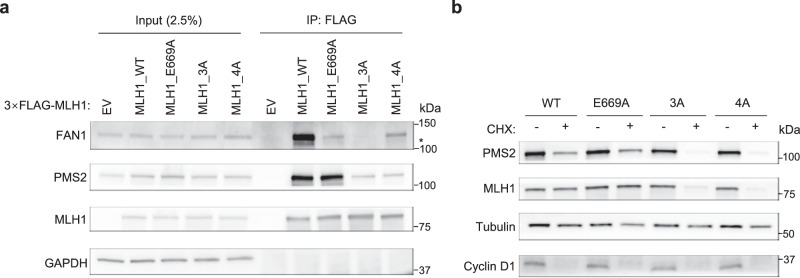
Mutation of essential MLH1 interaction residues impairs FAN1 binding in cells. **a** Immunoblots of FLAG co-immunoprecipitates from HEK293T transfected with either 3×FLAG-EV (empty vector) or the indicated 3×FLAG-MLH1 constructs (transfected at varying amounts to balance expression), along with PMS2. The asterisk (*) denotes signal bleed-through from overexpressed PMS2. **b** Immunoblots of HEK293T cell lysates with or without 8 h cycloheximide (CHX, 100 µg/mL) treatment, following transfection with either 3×FLAG-EV (empty vector) or the indicated 3×FLAG-MLH1 constructs together with PMS2. MLH1_3A: L585A/L636A/L653A; MLH1_4A: L574A/L639A/V647A/L650A. Data shown are representative of three independent experiments with similar results. Source data are provided as a Source Data file.

To assess the functional importance of key MLH1 residues for the MLH1–FAN1 interaction in a cellular context, we expressed FLAG-tagged wild-type or mutant MLH1 in MLH1-deficient HEK293T cells. Co-immunoprecipitation (co-IP) assays using anti-FLAG M2 affinity gel revealed that only wild-type MLH1 efficiently co-precipitates FAN1 (Fig. 6a). By contrast, the MLH1_E669A mutation, which disrupts the interaction with the FAN1-MIP motif, significantly weakens the association, and the MLH1_4A mutant, which perturbs the hydrophobic cage accommodating FAN1_I150 of the FAN1-MIM motif, also reduces binding. Notably, the MLH1_3A mutant, which impairs hydrophobic contacts with the C-terminal region of FAN1-MIM, completely abolishes FAN1 binding (Fig. 6a). Co-IP assays using an anti-FAN1 antibody corroborates the reduced interactions with MLH1_3A and, to a lesser extent, with MLH1_4A (Supplementary Fig. 7). These cellular findings align with prior studies identifying S126, Y128, F129, L155, and L159 of FAN1 as critical for the MLH1–FAN1 interaction^38,39^, and are consistent with our structural, ITC, and BLI data. Together, these results underscore the essential contributions of both MLH1 residues and the FAN1-MIP/MIM motifs to the MLH1–FAN1 interaction in cells.

We also observed that the MLH1_3A and MLH1_4A mutants exhibit lower expression and reduced binding to PMS2 (Fig. 6a), which corroborates cycloheximide (CHX) chase experiments indicating impaired MLH1 heterodimer stability for these mutants (Fig. 6b). This is not surprising, as both MLH1_3A and MLH1_4A mutants match (or are structurally adjacent to) previously reported Lynch syndrome variants^15,47,48^. Individual mutations such as L574P and L653R have been shown to reduce MLH1 (and PMS2) protein stability and impair MMR activity^49–51^. The destabilizing effect of these mutations on MLH1–PMS2 dimerization complicates further functional analysis of the MLH1–FAN1 interaction in the context of HD, as phenotypic outcomes could not be unambiguously attributed to loss of FAN1 binding. However, previous studies have shown that disrupting the MLH1–FAN1 interaction impairs the repair of CAG repeat expansion in HD^38,39^. Here, we uncover the structural basis of this specific interaction. In summary, our cellular co-IP experiments not only confirm that key MLH1 residues are critical for FAN1 binding, but also directly bridge our biophysical and structural data to a physiological context, thereby firmly establishing the molecular basis for the MLH1–FAN1 interaction.

## Discussion

**Fig. 7:**
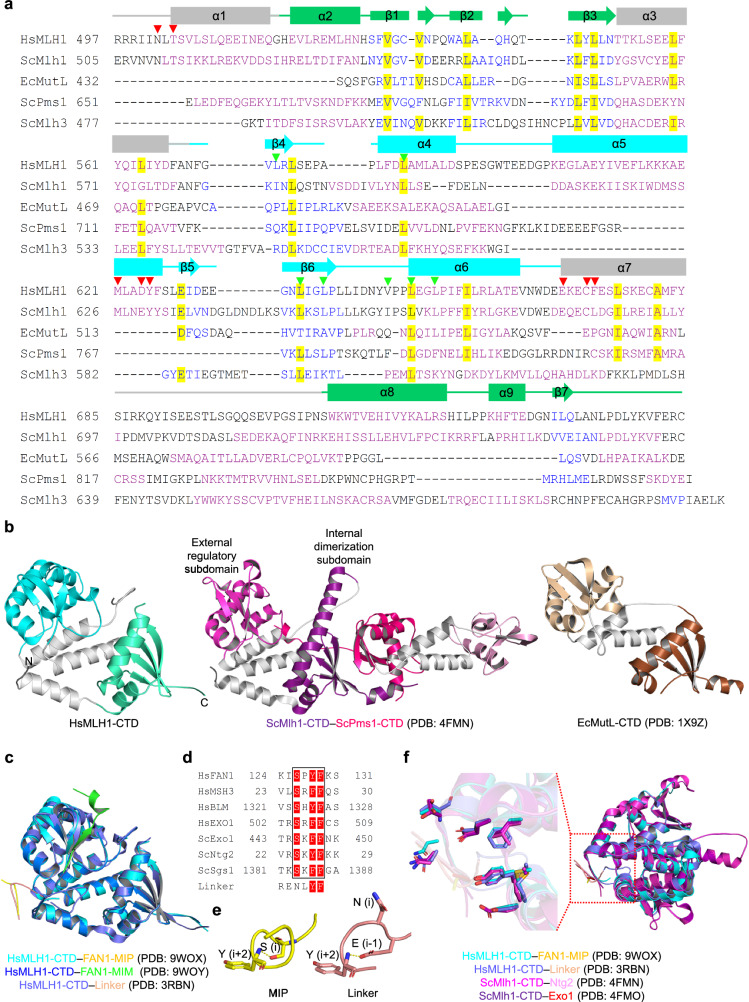
Structural comparison reveals a conserved protein–ligand recognition mechanism of MLH1-CTD–MIP-box motif. **a** Structure-based sequence alignment of solved CTDs of MLH1 homolog. Secondary structure elements of HsMLH1-CTD are indicated above the sequence alignment. The MIP and MIM interacting residues are marked by red and green triangles, respectively. The secondary elements of each sequence are colored in purple (for helices) and blue (for strands), with the highly conserved residues highlighted in yellow. **b** Overall structures of HsMLH1-CTD, ScMlh1-CTD–ScPms1-CTD heterodimer (PDB: 4FMN) and EcMutL-CTD (PDB: 1X9Z) shown in cartoon representation. The external regulatory subdomain and internal dimerization subdomain of HsMLH1-CTD, ScMlh1-CTD, ScPms1-CTD, EcMutL-CTD are colored in cyan, green-cyan, magenta, deep purple, pink, hot pink, wheat, and brown, respectively and the inter-subdomain linkers are colored in gray. **c** Superposition of HsMLH1-CTD–FAN1-MIP (PDB: 9WOX, this study) and HsMLH1-CTD–FAN1-MIM (PDB: 9WOY, this study) complexes on the apo structure of HsMLH1-CTD (PDB: 3RBN). **d** Sequence alignment of representative MIP-box motif from different species and the linker sequence in the MLH1-CTD apo structure (PDB: 3RBN). **e** ST-turn formed by intramolecular hydrogen bond by the side chain of serine (position i) and the main-chain amide group of tyrosine/phenylalanine (i + 2) in the MIP-box motif (left), and a variant turn formed by intramolecular hydrogen bond by the side chain of glutamic acid (i-1) and the main-chain amide group of tyrosine (i + 2) in the mimic MIP-box motif sequence of the linker in the MLH1-CTD apo structure (right). **f** Superposition of HsMLH1-CTD–FAN1-MIP complex (PDB: 9WOX, this study) on the apo structure of HsMLH1-CTD (PDB: 3RBN), ScMlh1-CTD–ScNtg2-MIP complex (PDB: 4FMN), and ScMlh1-CTD–ScExo1-MIP complex (PDB: 4FMO). Abbreviations: Hs, *Homo sapiens* (Human); Sc, *Saccharomyces cerevisiae* (Baker’s yeast); Ec, *Escherichia coli* (Bacteria).

Structure-based sequence alignment of MLH1-CTD with other MutL homologs revealed relatively low amino acid sequence conservation (Fig. 7a). Nevertheless, the overall tertiary structure of the CTD is highly conserved across homologs, consistently comprising an internal dimerization subdomain and an external regulatory subdomain (Fig. 7b). Superposition of the backbone structures of FAN1-MIP-bound MLH1-CTD (PDB: 9WOX, this study), FAN1-MIM-bound MLH1-CTD (PDB: 9WOY, this study), and apo MLH1-CTD (PDB: 3RBN) yields root mean square deviations less than 0.5 Å^52^, indicating that the binding of different peptides does not induce significant conformational changes in the protein backbone (Fig. 7c).

Previous studies have shown that *S. cerevisiae* Mlh1-CTD recognizes the MIP-box in the exonuclease Exo1, DNA N-glycosylase Ntg2, and DNA helicase Sgs1, and that human EXO1, DNA helicase BLM and MSH3 (a component of MutSβ) also interact with MLH1-CTD via the MIP-box motif^15,18,40^. Interestingly, in the apo MLH1-CTD structure, the linker sequence (GRENLYF) between the 6×His tag and MLH1-CTD occupies the FAN1-MIP binding site (Fig. 7c), suggesting that this linker may itself represent a MIP-box mimic. Sequence analysis revealed that the MIP-box sequence is highly conserved among MLH1-interacting proteins with an Sx(Y/F)F tetrapeptide motif (Fig. 7d). This motif adopts an ST-turn by the hydrogen bond between the side chain of serine (position i) and the main-chain amide of the tyrosine or phenylalanine at position i + 2 (Fig. 7e)^45^. The linker sequence GRENLYF, however, appears to be a MIP-box variant in which the serine is replaced by asparagine, and a distinct turn is formed via a hydrogen bond between the side chain of glutamic acid (i-1) and the main chain of tyrosine (i + 2) (Fig. 7e). Despite sequence and conformational variations, both canonical MIP-box motifs and the linker mimic bind to the same region, the S2 site of MLH1-CTD, engaging a conserved set of MLH1 residues in similar spatial orientations (Fig. 7f). Given that FAN1-MIP and MSH3-MIP share this S2 site, we performed competitive ITC assays, which confirmed that FAN1-MIP competes with MSH3-MIP for MLH1 binding (Supplementary Fig. 3). This competition suggests a mechanism by which FAN1 may block the interaction between MLH1 and MSH3, consistent with earlier reports that FAN1 inhibits MMR-driven CAG repeat expansion by sequestering MLH1 from MSH3^38^. Collectively, our structural comparisons support a conserved molecular mechanism for MLH1–MIP-box recognition across species and interaction partners.

**Fig. 8:**
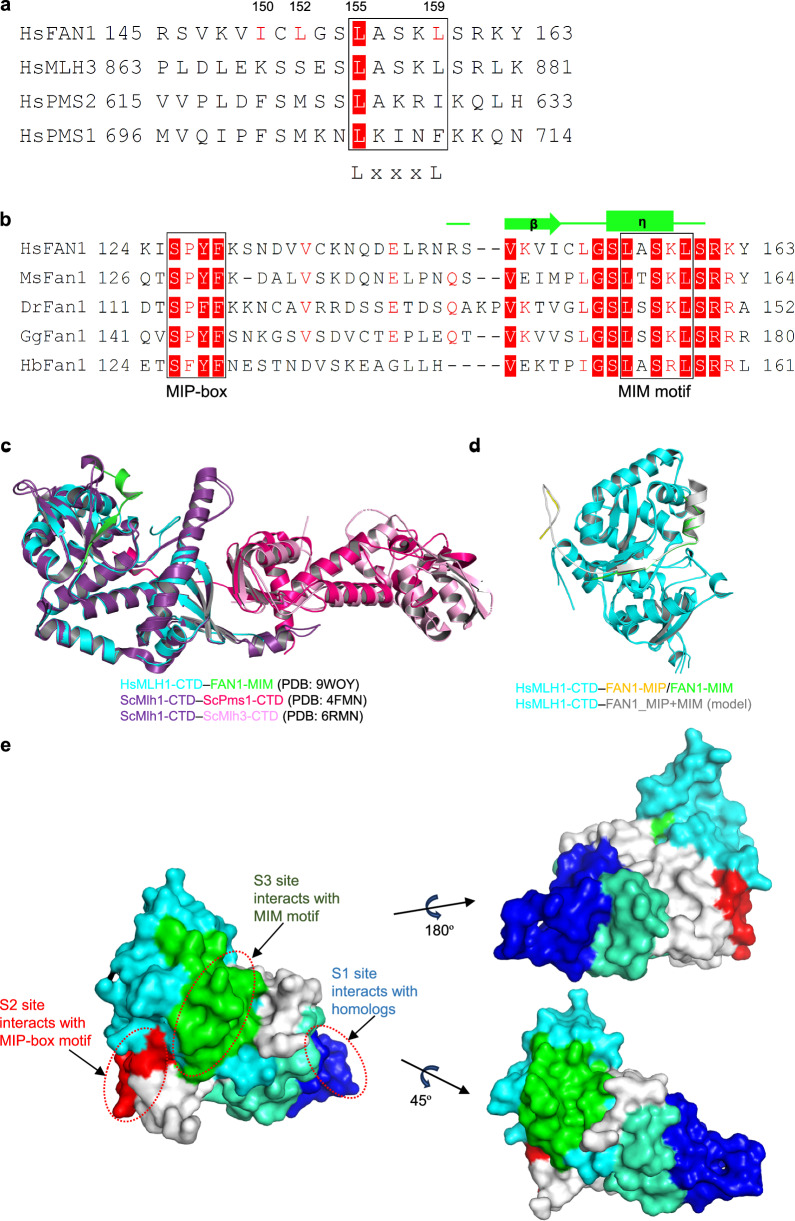
Structural comparison identifies an unrecognized binding site on MLH1-CTD engaged for FAN1-MIM interaction. **a** Sequence alignment of the FAN1-MIM and putative MIM motif in HsMLH1 homologs. The highly conserved leucine and the HsMLH1-CTD hydrophobic interaction residues are highlighted in red. **b** Sequence alignment of the FAN1 fragment encompassing the MIP-box and MIM motif across different species. Secondary structure elements of HsFAN1-MIM are indicated above the sequence alignment. **c** Superposition of HsMLH1-CTD–FAN1-MIM complex (PDB: 9WOY, this study) on the ScMlh1-CTD–ScPms1-CTD complex (PDB: 4FMN), and ScMlh1-CTD–ScMlh3-CTD complex (PDB: 6RMN). **d** Superposition of HsMLH1-CTD–FAN1-MIP and HsMLH1-CTD–FAN1-MIM complexes on the predicted HsMLH1-CTD–FAN1-MIP + MIM (residues 124–163) complex. **e** Surface representation of MLH1-CTD, with the S1, S2, S3 sites colored in blue, red and green, and the regulatory subdomain, dimerization subdomain and inter-subdomain linker colored in cyan, green-cyan, gray, respectively. Abbreviations: Hs, *Homo sapiens* (Human); Ms, *Mus musculus* (Mouse); Dr, *Danio rerio* (Zebrafish); Gg, *Gallus gallus* (Chicken); Hb, *Hymenochirus boettgeri* (Congo dwarf clawed frog); Sc, *Saccharomyces cerevisiae* (Baker’s yeast).

Previous study discovered a 36-amino acid region in MLH1 homologs MLH3, PMS2, and PMS1 that mediates their interaction with MLH1^53^. Subsequently, Porro et al. identified a highly conserved 12-residue sequence downstream of the FAN1-MIP box, which exhibits extensive homology with part of this 36-amino acid sequence, and named it MIM^39^. The MIM motif consists of a conserved LxxxL pentapeptide motif (Fig. 8a), which is well conserved in FAN1 proteins across different species (Fig. 8b). The MLH1-CTD–FAN1-MIM complex crystal structure obtained by us indicates that four residues within this LxxxL pentapeptide motif in MIM are involved in forming a 3_10_ helix (η, S154–K158, Figs. 4 and 8b). The side chains of the first leucine (L155), the second leucine (L159), and a preceding leucine (L152) of this motif engage in extensive hydrophobic interactions with MLH1 residues L585, L636, and L653 (Fig. 4 and Supplementary Fig. 6a). Additionally, our complex structure reveals that a β-strand (β, V147–I150) preceding the LxxxL motif runs parallelly and forms numerous intermolecular hydrogen bonds with MLH1-β4, and the side chain of FAN1 residue I150 inserts into a hydrophobic pocket composed of MLH1 residues L574, L639, V647, and L650 (Fig. 4 and Supplementary Fig. 6b). All of these interactions further stabilize the interaction between FAN1-MIM and MLH1-CTD. Sequence alignments indicate that most of these above-mentioned hydrophobic residues are conserved in FAN1 orthologs (Fig. 8b). Additionally, the intermolecular hydrogen bonds between FAN1 and MLH1 are conducted by main chains, suggesting that this interaction mode should be conserved in different species. Furthermore, the MIP-box motif upstream of the MIM motif is also widely conserved among FAN1 proteins (Fig. 8b), supporting the notion that the interaction between FAN1 and MLH1 should be conserved across different species.

Structural comparison with the CTD heterodimers of Mlh1–Pms1 and Mlh1–Mlh3 from *S. cerevisiae* uncovered that the FAN1-MIM binds to a previously uncharacterized region of the MLH1-CTD (Fig. 8c). Following existing nomenclature, we term this region the S3 site^15–18^. This finding indicates that FAN1 binding does not sterically interfere with MLH1 heterodimerization with its homologous partners such as PMS2 or MLH3, and vice versa. Consistent with this structural observation, previous studies have shown that MLH1 efficiently co-precipitates PMS2 in cells expressing FAN1^38^, and that recombinant GST-FAN1 effectively pulls down recombinant MutLα (MLH1–PMS2) and MutLγ (MLH1–MLH3)^39^, demonstrating that the MLH1–FAN1 interaction can occur in the context of pre-formed MutL heterodimers.

Although we were unable to determine the crystal structure of MLH1-CTD in complex with the full FAN1-MIP + MIM peptide (residues 124–163), we generated a structural model using AlphaFold Multimer (Fig. 8d)^54^. This model corroborates our crystal structures, showing that the FAN1-MIP + MIM fragment simultaneously engages the S2 and S3 sites of MLH1-CTD via its MIP-box and MIM motif, respectively. Notably, a flexible loop connecting the MIP and MIM motifs of FAN1 may account for the difficulty in obtaining crystals of the MLH1-CTD and FAN1-MIP + MIM peptide complex (Fig. 8d). Taken together, these findings demonstrate that MLH1 engages heterodimer partners, MIP-box-containing proteins, and MIM-motif-bearing proteins through its S1, S2, and S3 regions, respectively (Fig. 8e). Collectively, our work identifies an undiscovered binding site on MLH1 for the FAN1-MIM, revealing an interaction mode that appears to be conserved across species.

A related question concerns whether MLH1 heterodimerization with its canonical partners (PMS2, MLH3) influences its ability to engage FAN1. Our structural analysis shows that the FAN1-binding S2 and S3 sites are spatially separated from the S1 dimerization interface (Figs. 4f and 8e), and previous co-precipitation studies have demonstrated that FAN1 can interact with MutLα and MutLγ complexes^38,39^, indicating that dimerization does not preclude FAN1 binding. However, whether heterodimer formation quantitatively modulates the affinity of the MLH1–FAN1 interaction remains to be determined. Direct comparative binding measurements using purified MLH1–PMS2 heterodimer versus MLH1-CTD alone would be required to address this question definitively, representing an important direction for future investigation.

In summary, our quantitative ITC experiments illustrate that MLH1-CTD binds to the isolated FAN1-MIP and FAN1-MIM motifs with comparable affinities, and that their tandem arrangement significantly enhances the overall interaction. We further demonstrate that FAN1-MIP competes with MSH3-MIP for the shared binding site (S2) on MLH1, a mechanism that likely blocks the MutL–MutSβ interaction and thereby suppresses the CAG repeat expansion, consistent with earlier reports^38^. From a structural perspective, we show that FAN1-MIP recognition follows a conserved mechanism, whereas the FAN1-MIM engages the previously uncharacterized S3 site on MLH1-CTD through extensive hydrophobic and hydrogen-bonding interactions. This S3-binding mode is distinct from the known S1 (dimerization) and S2 (MIP-binding) sites within MLH1-CTD, underscoring the MLH1-CTD as a versatile protein interaction hub (Fig. 8e). Additionally, these structural insights suggest that the MLH1–FAN1 interaction does not impede MutL heterodimer formation but specifically interferes with the interaction between MutL and MutSβ. Co-immunoprecipitation assays further confirm that mutation of the key MLH1 residues disrupts FAN1 binding in a cellular environment. Together with previous functional studies^38,39^, our work provides a structural elucidation of how the human MLH1-CTD recognizes the FAN1-MIP and FAN1-MIM motifs. This establishes a crucial structural framework for understanding the molecular mechanism by which this interaction regulates CAG trinucleotide repeat expansion in HD.

## Methods

### Protein expression and purification

DNA fragments encoding MLH1-CTD (residues 486–751 or 495–751) and FAN1 (residues 118–177) were subcloned into a pET28-MHL vector (Addgene, 26096) to generate N-terminal His-TEV-tagged fusion proteins. All the plasmids were constructed using a seamless assembly cloning kit (ABclonal Technology, RK21020) and verified by Sanger sequencing (Azenta Life Sciences). Recombinant proteins were overexpressed in *E. coli* BL21 (DE3) Codon plus RIL (TransGen, CD601) at 15 °C for 24 h under induction with 0.25 mM IPTG (isopropyl-β-D-thiogalactoside) at an OD₆₀₀ value of 0.8. Cells were harvested, resuspended and lysed by sonication with the ultrasonic power set to 25%, and the ultrasonic disruption mode of 3-s on, 7-s off for 15 min and 2–3 cycles. After cell lysis and centrifugation (45 min at 25,000 × g), the supernatant was subjected to an open column with Ni-nitrilotriacetate resin (GE Healthcare, 17526802) for affinity purification followed by TEV (tobacco etch virus) protease treatment to remove the His-tag. The resultant protein was further purified by size-exclusion chromatography on a Superdex200 gel-filtration column (GE Healthcare, 28989335) for MLH1-CTD or Superdex75 gel-filtration column (GE Healthcare, 28989333) for FAN1 in a buffer containing 20 mM Tris-HCl, pH 7.5, 150 mM NaCl, and 1 mM DTT. Purified proteins were concentrated up to 25 mg/mL using Amicon Ultra-15 Centrifugal Filter Units (Millipore, UFC901024), aliquoted, and stored at –80 °C for future use. The buffer conditions for Ni-affinity chromatography were as follows: lysis buffer: 20 mM Tris-HCl, pH 7.5, 250 mM NaCl, 5% glycerol, and 5 mM β-mercaptoethanol; wash buffer: 20 mM Tris-HCl, pH 7.5, 1 M NaCl, and 40 mM imidazole; elution buffer: 20 mM Tris-HCl, pH 7.5, 250 mM NaCl, and 250 mM or 1 M imidazole.

For co-expression of MLH1-CTD and FAN1, the DNA sequences encoding FAN1 (residues 118–177) and MLH1-CTD (residues 495–751) were cloned into the first and second multiple cloning sites of pETDuet-1-LIC vector (www.thesgc.org), respectively, to express His-TEV-tagged fusion FAN1 protein and tag-free MLH1-CTD. The co-expressed FAN1 and MLH1-CTD complex protein was purified by Ni-affinity chromatography followed by Superdex200 size-exclusion chromatography.

All the point mutations were introduced using Fast Mutagenesis System Kit (Transgene, FM111-02) according to manufacturer’s instruction and confirmed by Sanger sequencing (Azenta Life Sciences). Mutant proteins were expressed and purified using the same procedures as for the wild-type constructs. All the primers used in this study were provided in Supplementary Table 2.

### Isothermal titration calorimetry (ITC)

For the ITC measurement, concentrated proteins were diluted into the ITC buffer (20 mM Tris-HCl, pH 7.5, 150 mM NaCl), and lyophilized peptides (Shanghai Apeptide Co., Ltd or GLS GL Biochem (Shanghai) Ltd.) were dissolved in the same buffer, with pH adjusted by adding 2 M NaOH. Peptide concentrations were estimated based on their mass. All the measurements were performed in duplicate at 25 °C utilizing an iTC-200 (MicroCal, Inc.) microcalorimeter. The procedure is as following: the protein with a concentration of 50 µM was loaded in the cell chamber, and peptide or protein with a concentration of 750 µM in the syringe was injected into the cell for 20 successive injections with a spacing of 150 s between injections. Control experiments were carried out under identical conditions to determine the heat signals that resulted from injecting peptides or proteins into the buffer. Data were analyzed using the single-site binding model after subtraction of the control heat signals within the Origin software package (MicroCal, Inc.).

Competitive ITC assays were performed as previously described^55^. Briefly, MLH1-CTD protein was pre-incubated with a 1:1 molar ratio of either FAN1-MIP or MSH3-MIP peptide on ice for 30 min. The resulting protein–peptide mixture was then titrated with the competing peptide MSH3-MIP or FAN1-MIP, respectively, following the procedure described above.

### Biolayer interferometry (BLI)

For the BLI measurement, MLH1-CTD (10 mg/mL) was buffer-exchanged into PBS and biotinylated using a Biotinylation Kit (BMD Labservice, G-MM-IGT). The FAN1 peptides were dissolved in PBST (PBS with 0.05% Tween 20) with a concentration of 10 mM and serially diluted in PBST for binding assays. All the assays were run at 30 °C with continuous shaking at 1000 rpm using an Octet-R2 system (Sartorius). Before each assay, the streptavidin (SA) biosensors were pre-wetted in PBST to record the baseline. The biotinylated MLH1-CTD protein was immobilized onto the SA biosensor through three steps: baseline, loading, and baseline again, to obtain the sample biosensor. Then the sample biosensor (immobilized with biotinylated MLH1-CTD) and the control blank biosensor were incubated with FAN1 peptide from lower to higher concentrations by three steps: (1) 60-s baseline acquisition, (2) 60-s association of FAN1 peptide for the measurement of *k*_on_, and (3) 120-s dissociation of FAN1 peptide for the measurement of *k*_off_. The BLI assays of the wild-type and mutant MLH1-CTD with C-terminal biotin labeled FAN1 peptide (residues 145–163) were conducted using the same procedure with minor modifications: (1) 60-s baseline acquisition, (2) 180-s association of MLH1-CTD protein for the measurement of *k*_on_, and (3) 200-s dissociation of MLH1-CTD protein for the measurement of *k*_off_. For the BLI assays with MBP-tag fused MLH1-CTD protein, the control biosensor was immobilized with biotinylated MBP alone. Raw kinetic data were collected using Data Acquisition software (Octet BLI Discovery 13.0) and association/dissociation rate constants (*k*_on_/*k*_off_) were generated with the reference subtraction method using Data Analysis software (Octet Analysis Studio 13.0), and affinity (*K*_D_) was calculated accordingly.

### Protein crystallization

For crystallization of the MLH1-CTD–FAN1 peptide complexes, purified MLH1-CTD (10 mg/mL) was mixed with the respective FAN1 peptide at a molar ratio of 1:3. Then, the mixture was crystallized using the sitting-drop vapor diffusion method at 18 °C by adding 0.5 μL of the protein–peptide mixture with 0.5 μL of the reservoir solution. The MLH1-CTD–FAN1-MIP complex was crystallized in a reservoir solution containing 0.2 M lithium sulfate monohydrate, 0.1 M Tris-HCl, pH 8.5, 30% w/v polyethylene glycol 4000. The MLH1-CTD–FAN1-MIM complex was crystallized in a reservoir solution consisting of 0.2 M trimethylamine N-oxide dihydrate, 0.1 M Tris-HCl, pH 8.5, and 20% w/v polyethylene glycol monomethyl ether 2000. Prior to flash-freezing in liquid nitrogen, crystals were cryoprotected by soaking in a solution containing 85% reservoir solution and 15% glycerol.

### Data collection and structure determination

The diffraction data of the crystals were collected at beamline BL10U2 of the Shanghai Synchrotron Radiation Facility (SSRF) at 100 K. Diffraction images were processed using autoPROC^56^/AIMLESS^57^. The structures were solved by molecular replacement with PHASER^58^ using the crystal structure of MLH1-CTD (PDB: 3RBN) as the search model. For both crystal structures, interactive model was built with COOT^59^, followed by initial restrained refinement with REFMAC^60^. The model was then automatically rebuilt with ARP/wARP^61^, further refinement with PHENIX^62^ and REFMAC. Restrained refinement and validation were performed with MOLPROBITY^63^. Crystal diffraction data and refinement statistics for the structures were presented in Supplementary Table 1.

### Cell culture

HEK293T cells (ATCC, CRL-11268) were maintained in Dulbecco’s modified Eagle’s medium (DMEM; Thermo Fisher Scientific, 41966029) supplemented with 10% fetal calf serum (FCS; Thermo Fisher Scientific, A5256701) and 1% penicillin–streptomycin (Thermo Fisher Scientific, 15140-122).

### Transient transfection

HEK293T cells were transiently transfected with either 3×FLAG-empty vector (EV) or 3×FLAG-MLH1 (at varying amounts), together with PMS2 (1 µg per 10 cm dish) constructs using Lipofectamine 2000, following manufacturer’s instruction. To achieve comparable expression levels of 3×FLAG-MLH1 across mutants, different amounts of plasmid were transfected (per 10 cm dish): 0.5 µg for MLH1_WT, 1 µg for MLH1_E669A, and 5 µg each for MLH1_3A (L585A/L636A/L653A) and MLH1_4A (L574A/L639A/V647A/L650A). The total DNA amount was kept constant by supplementing with 3×FLAG-EV. Cells were harvested 48 h post-transfection for co-immunoprecipitation or cycloheximide chase assays.

### Co-immunoprecipitation (co-IP)

Forty-eight hours after transfection, cells were harvested, washed with ice-cold PBS, and scraped in NP-40 lysis buffer (50 mM Tris-HCl, pH 7.5, 120 mM NaCl, 1 mM EDTA, 6 mM EGTA, 15 mM sodium pyrophosphate, 20 mM sodium fluoride, 1% NP-40) supplemented with phosphatase and protease inhibitors (5 mM sodium fluoride, 1 mM PMSF, 0.5 mM sodium orthovanadate, and 1×EDTA-free cOmplete protease inhibitor cocktail (Roche, 11873580001)). Lysates were snap-frozen, thawed on ice, and passed through a 22 G needle (Braun), followed by incubation with Benzonase (500 U/mL, Merck, 1016540001) for 2 h at 4 °C. Cleared lysates were obtained by centrifugation at 14,909 × g for 13 min at 4 °C. Protein concentrations were determined, and 2 mg of lysate was incubated with 20 µL anti-FLAG M2 affinity gel (Sigma-Aldrich, A2220) overnight at 4 °C for FLAG co-IP. For FAN1 co-IP, 2 mg of lysate was incubated with 2 µg FAN1 antibody (Proteintech, 17600-1-AP, 1:1000) for 2 h at 4 °C. Meanwhile, Protein A/G Magnetic Agarose beads (Thermo Scientific, 78609) were equilibrated with NP-40 buffer. The lysate–antibody mixture was added to the beads and incubated for 2 h at 4 °C. The beads were washed thrice with NP-40 buffer and once with TEN100 (20 mM Tris-HCl, pH 7.4, 0.1 mM EDTA, 100 mM NaCl). Immunoprecipitated complexes were eluted by boiling in SDS sample buffer, resolved by SDS-PAGE, and transferred to nitrocellulose membranes.

Membranes were incubated overnight at 4 °C with the appropriate primary antibodies: rabbit anti-FAN1 (Proteintech, 17600-1-AP, 1:1000), rabbit anti-MLH1 (Abcam, ab92312 (EPR3895), 1:500), rabbit anti-PMS2 (Abcam, ab110638 (EPR3947), 1:10,000), mouse anti-GAPDH (Millipore, MAB374 (6C5), 1:20,000), mouse anti-tubulin (Sigma-Aldrich, T9026 (DM1A), 1:20,000). After washing, membranes were incubated for 1 h at room temperature with species-appropriate HRP-conjugated secondary antibodies (donkey anti-rabbit, Cytiva, NA934, 1:5000; sheep anti-mouse, Cytiva, NA931, 1:5000). Proteins were detected using Advansta WesternBright ECL reagent (Advansta, K-12043-D10) and visualized on a Fusion Solo S imaging system.

### Cycloheximide chase experiments

At 48 h post-transfection, cells were treated with 100 µg/mL cycloheximide (CHX) or left untreated for 8 h at 37 °C. Cells were then washed three times with PBS and scraped in Laemmli buffer (4% SDS, 20% glycerol, 120 mM Tris-HCl, pH 6.8). Lysates were boiled, sonicated, and quantified. Proteins were separated by SDS-PAGE, transferred to nitrocellulose membranes, and probed with the following primary antibodies overnight at 4 °C: rabbit anti-MLH1 (Abcam, ab92312 (EPR3895), 1:500), rabbit anti-PMS2 (Abcam, ab110638 (EPR3947), 1:10,000), mouse anti-tubulin (Sigma-Aldrich, T9026 (DM1A), 1:20,000), and rabbit anti-cyclin D1 (Cell Signaling, 2922, 1:1000). After washing, HRP-conjugated secondary antibodies were applied for 1 h at room temperature, and signals were detected as described above. The uncropped and unprocessed versions of blots generated in this study are provided in the Source Data file.

### Reporting summary

Further information on research design is available in the Nature Portfolio Reporting Summary linked to this article.

## Supplementary information

Supplementary Information

Reporting Summary

Transparent Peer Review file

## Source data

Source Data

## Data Availability

The atomic coordinates and structure factors generated in this study have been deposited in the Protein Data Bank (PDB) with accession codes 9WOX for MLH1-CTD–FAN1-MIP complex structure and 9WOY for MLH1-CTD–FAN1-MIM complex structure, respectively. The structural data used in this study are available in the PDB under accession codes 3RBN (MLH1-CTD apo structure), 4FMN^15^ (ScMlh1-CTD–ScPms1-CTD–ScNtg2-MIP complex), 4FMO^15^ (ScMlh1-CTD–ScPms1-CTD–ScExo1-MIP complex), 6RMN^16^ (ScMlh1-CTD–ScMlh3-CTD complex), 1X9Z^42^ (EcMutL-CTD), respectively. Source data are provided with this paper.
