# Supplementary material for: Structural insights into the MLH1–FAN1 interaction reveal an uncharacterized binding interface on MLH1
Source: Nat Commun. 2026 Jul 6;17:8367. doi: 10.1038/s41467-026-74991-0 (PMC13473659; doi:10.1038/s41467-026-74991-0)
Supplement: Supplementary file 1 [file 41467_2026_74991_MOESM1_ESM.pdf]

Supplementary Information

Structural insights into the MLH1–FAN1 interaction reveal an uncharacterized binding interface on MLH1

Yichang Chen[1, #], Haiyun Hu[1, #], Xinci Shang[1, #], Keri M. Fishwick[2], Giada Greco[2], Qin Xiao[1], Yinhao Zhou[1], Qiuyan Huang[1], Tao Jiang[1], Xiaolei Huang[1], Guanghui Wang[1], Xuechu Zhen[1], Guoqiang Xu[1], Su Qin[3], Alessandro A. Sartori[2] and Yanli Liu[1, 4, 5 *]

[1] Jiangsu Key Laboratory of Drug Discovery and Translational Research for Brain Diseases, College of Pharmaceutical Sciences, Soochow University, Suzhou, Jiangsu 215123, China.

[2] Institute of Molecular Cancer Research, University of Zurich, Strickhofstrasse 40a, CH-8057 Zurich, Switzerland.

[3] Life Science Research Center, Southern University of Science and Technology, Shenzhen, Guangdong 518055, China.

[4] Jiangsu Province Engineering Research Center of Precision Diagnostics and Therapeutics Development, Soochow University, Suzhou, Jiangsu 215123, China.

[5] Suzhou International Joint Laboratory for Diagnosis and Treatment of Brain Diseases, Suzhou, Jiangsu 215123, China.

[#] These authors contributed equally to this work. * To whom correspondence should be addressed. E-mail: ylliu18@suda.edu.cn

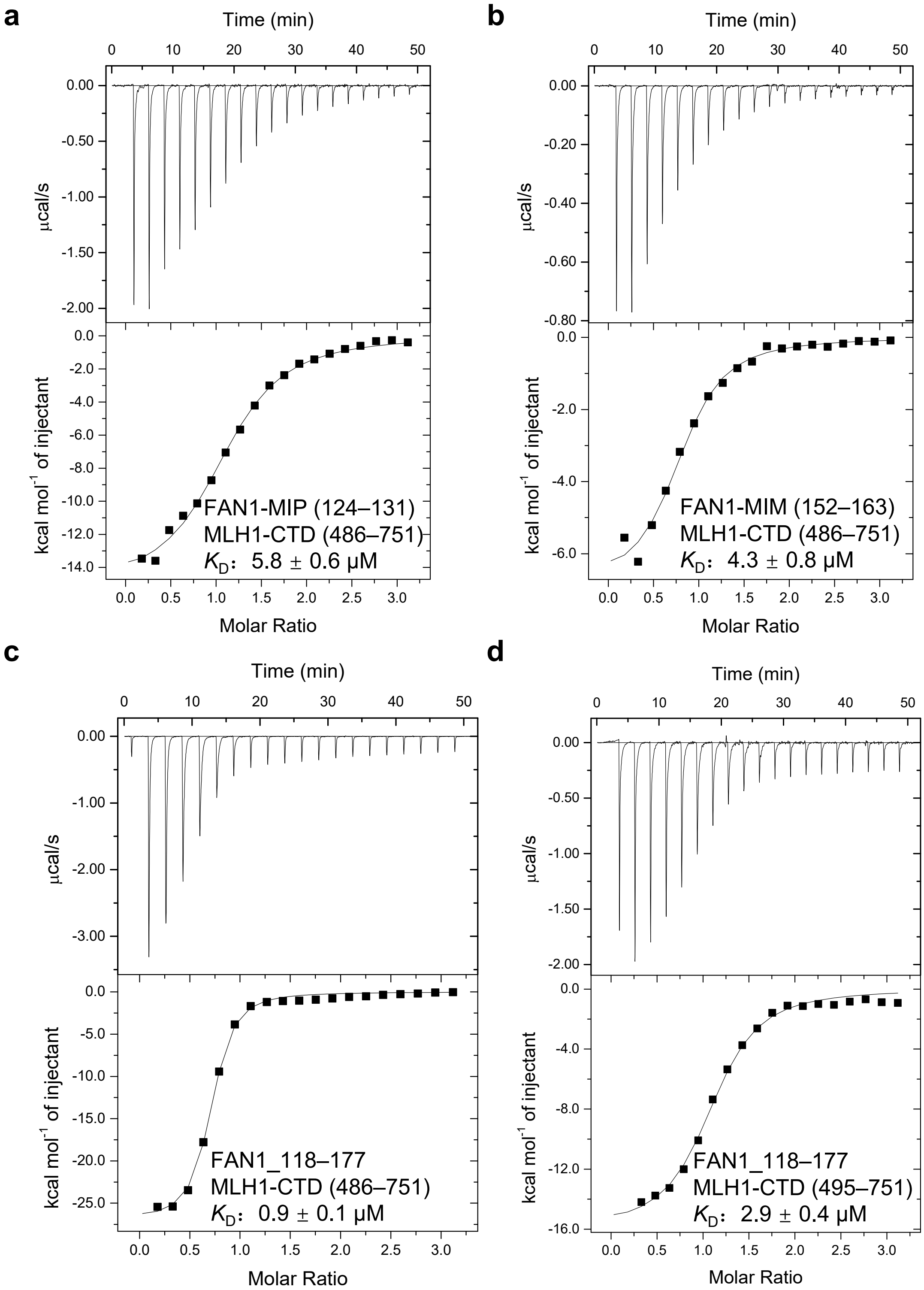

Supplementary Fig. 1 ITC binding curves for the titration of FAN1 peptides or recombinant FAN1 protein to recombinant MLH1-CTD proteins by iTC-200 microcalorimeter (MicroCal, Inc.). **a, b** FAN1-MIP **(a)** and FAN1-MIM **(b)** peptides titrate to MLH1-CTD protein (residues 486–751), respectively. **c, d** Recombinant FAN1 protein (residues 118–177) titrates to recombinant MLH1-CTD protein (residues 486–751) **(c)** and MLH1-CTD protein (residues 495–751) **(d)**, respectively. K_D: dissociation constant (μM); ITC data shown are representative of two independent experiments performed by iTC-200 microcalorimeter (MicroCal, Inc.).

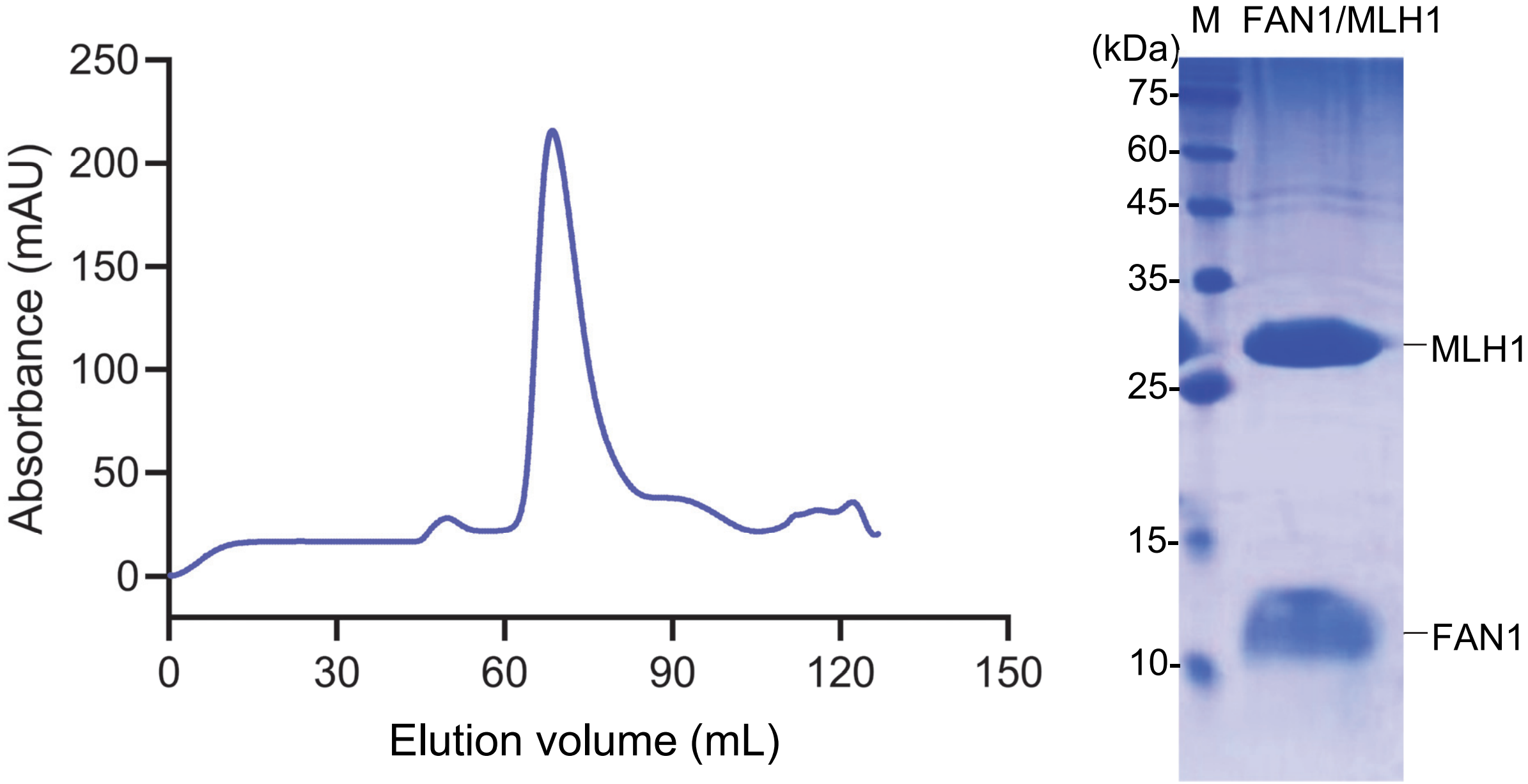

Supplementary Fig. 2 Co-expression and purification of MLH1-CTD and FAN1. The coding regions of MLH1-CTD (residues 495–751) and FAN1 (residues 118–177) were subcloned into pETDuet-1-LIC vector to express N-terminal 6×His tagged FAN1 and tag-free MLH1. The complex protein was purified by Ni^{2+} affinity chromatography and Superdex200 gel filtration.

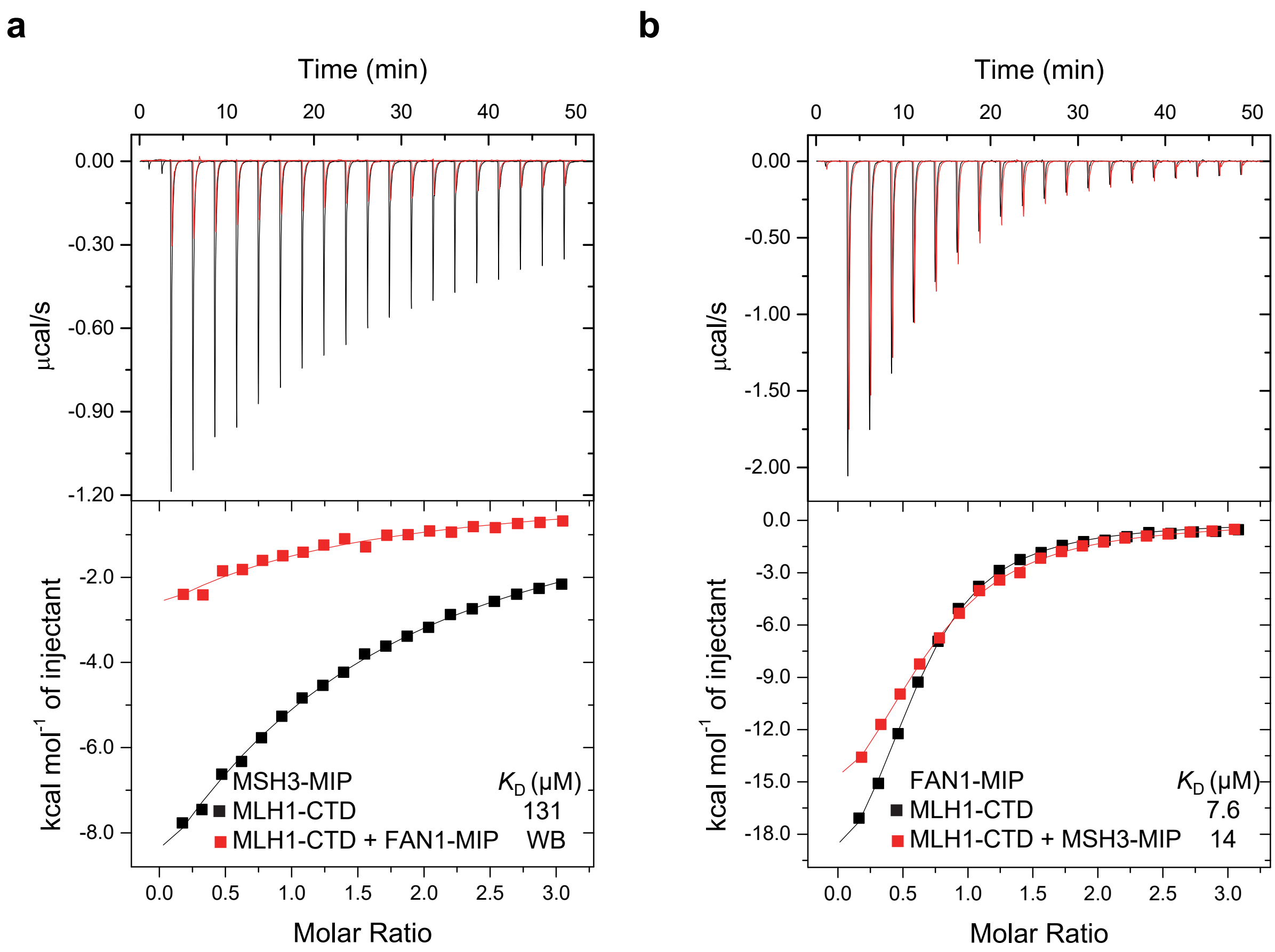

Supplementary Fig. 3 FAN1-MIP competes with MSH3-MIP for the binding site on MLH1-CTD. a Pre-incubation of MLH1-CTD with FAN1-MIP peptide weakens the interaction of MSH3–MLH1. ITC binding curves for the titration of MSH3-MIP peptide to MLH1-CTD alone or a mixture of MLH1-CTD with FAN1-MIP peptide at a molar ratio of 1:1 using an iTC-200 microcalorimeter (MicroCal, Inc.). **b** Pre-incubation of MLH1-CTD with MSH3-MIP peptide shows negligible effect on the interaction of FAN1–MLH1. ITC binding curves for the titration of FAN1-MIP peptide to MLH1-CTD alone or a mixture of MLH1-CTD with MSH3-MIP peptide at a molar ratio of 1:1 using an iTC-200 microcalorimeter (MicroCal, Inc.). K_D: dissociation constant (μM); WB: weak binding; ITC data shown are representative of two independent experiments performed by iTC-200 microcalorimeter (MicroCal, Inc.).

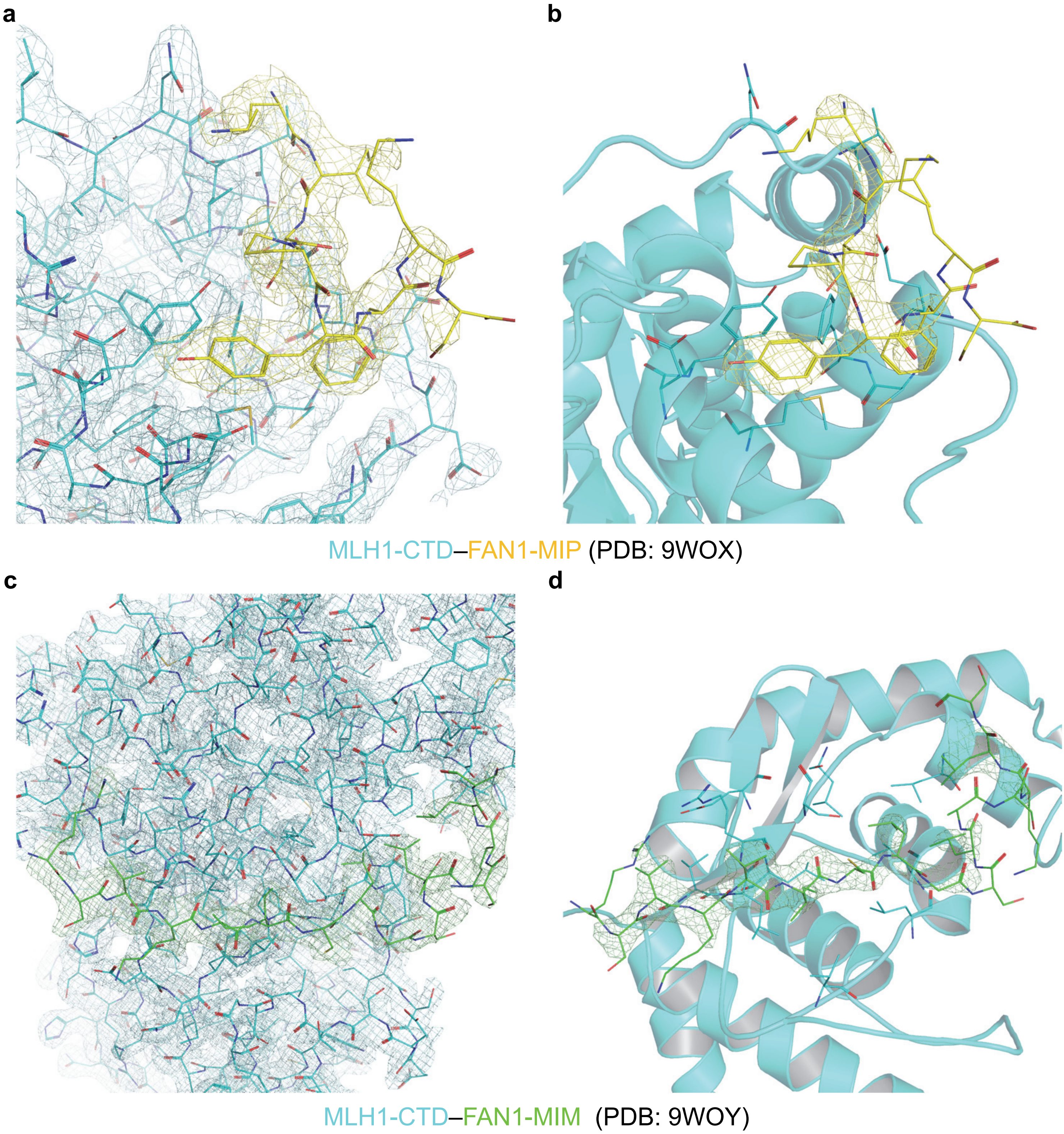

Supplementary Fig. 4 Portion of the electron density map and omit map of different complexes. **a, c** 2*Fo*-*Fc* electron density map contoured at 1σ. **b, d** *Fo*-*Fc* omit map contoured at 3σ.

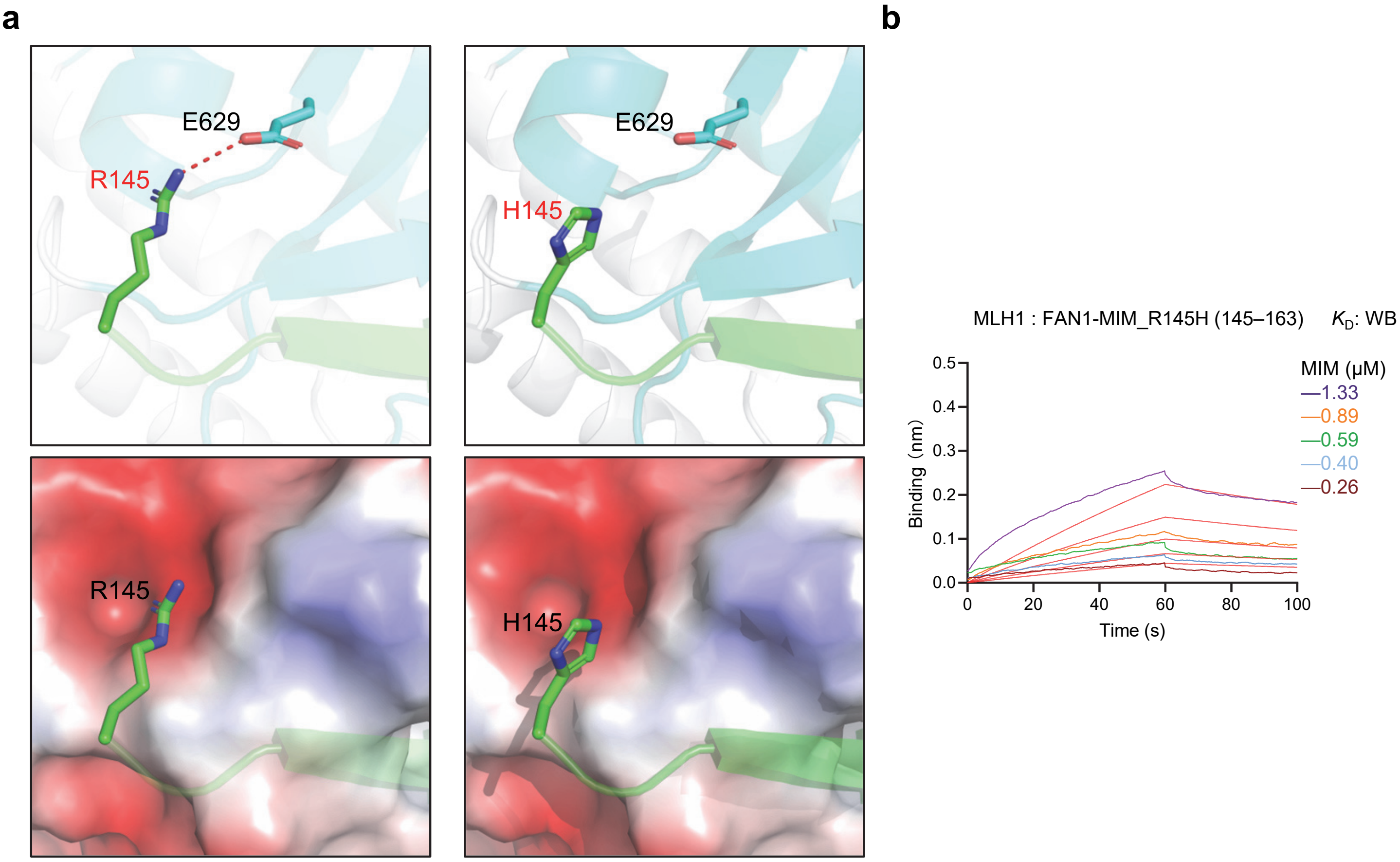

Supplementary Fig. 5 FAN1 variant R145H weakens the MLH1–FAN1 interaction. a Structural comparison of FAN1-R145 (left) and FAN1-R145H variant (right). **b** BLI binding curves for biotinylated MLH1-CTD (residues 495–751) with FAN1-MIM_R145H (145–163). WB: weak binding. Data shown are representative of two independent experiments. Source data are provided as a Source Data file.

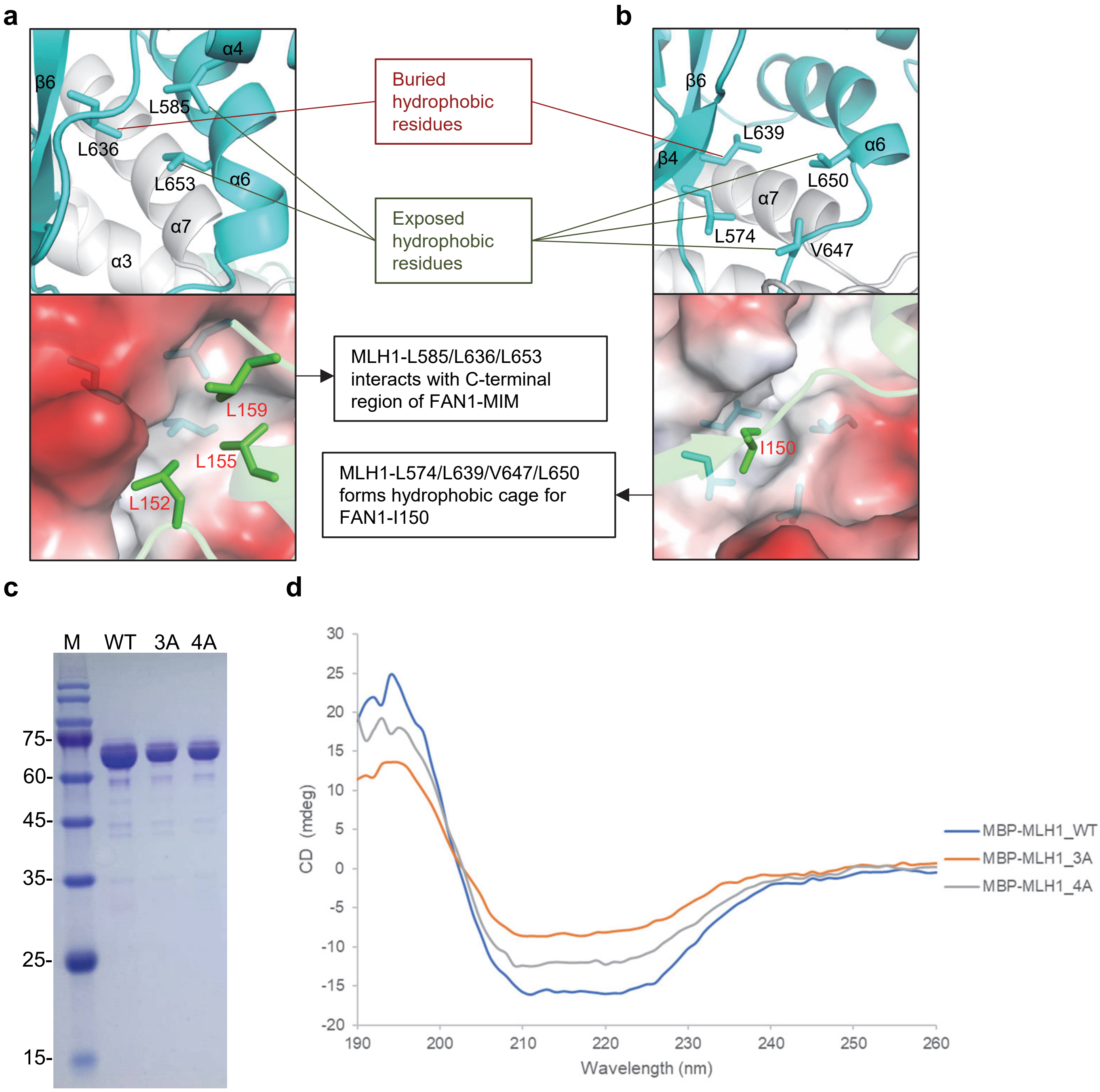

Supplementary Fig. 6 The MBP-tag fused MLH1-CTD multiple points mutant proteins could be expressed and folded properly in *E. coli* expression system. a, b The locations of the mutated residues in MLH1_3A **(a)** and MLH1_4A **(b)** with their solvent accessibility indicated. The hydrophobic patch for FAN1-MIM C-terminus **(a)** and the FAN1-I150 hydrophobic cage **(b)** shown as electrostatic potential surface representation. **c** The SDS-PAGE of purified MBP-MLH1_WT, MBP-MLH1_3A, MBP-MLH1_4A proteins. **d** Circular dichroism (CD) spectra of purified MBP-MLH1_WT, MBP-MLH1_3A, MBP-MLH1_4A proteins. Secondary structures of purified proteins were estimated from CD spectra on a J-1500 CD spectrophotometer (Jasco, Japan). The purified proteins were diluted in distilled water with a final concentration of 0.1 mg/mL and pipetted into a quartz optical cuvette with a path length of 1 cm. The spectra were scanned three times consecutively and averaged with a wavelength range 190–260 nm at 25 °C and bandwidth 1 nm. The signal of the solvent was subtracted to obtain the final signal of the samples. MBP-MLH1_3A: MBP-tag fused MLH1-CTD with L585A/L636A/L653A; MBP-MLH1_4A: MBP-tag fused MLH1-CTD with L574A/L639A/V647A/L650A. Data shown are representative of two independent experiments in **c**, **d**.

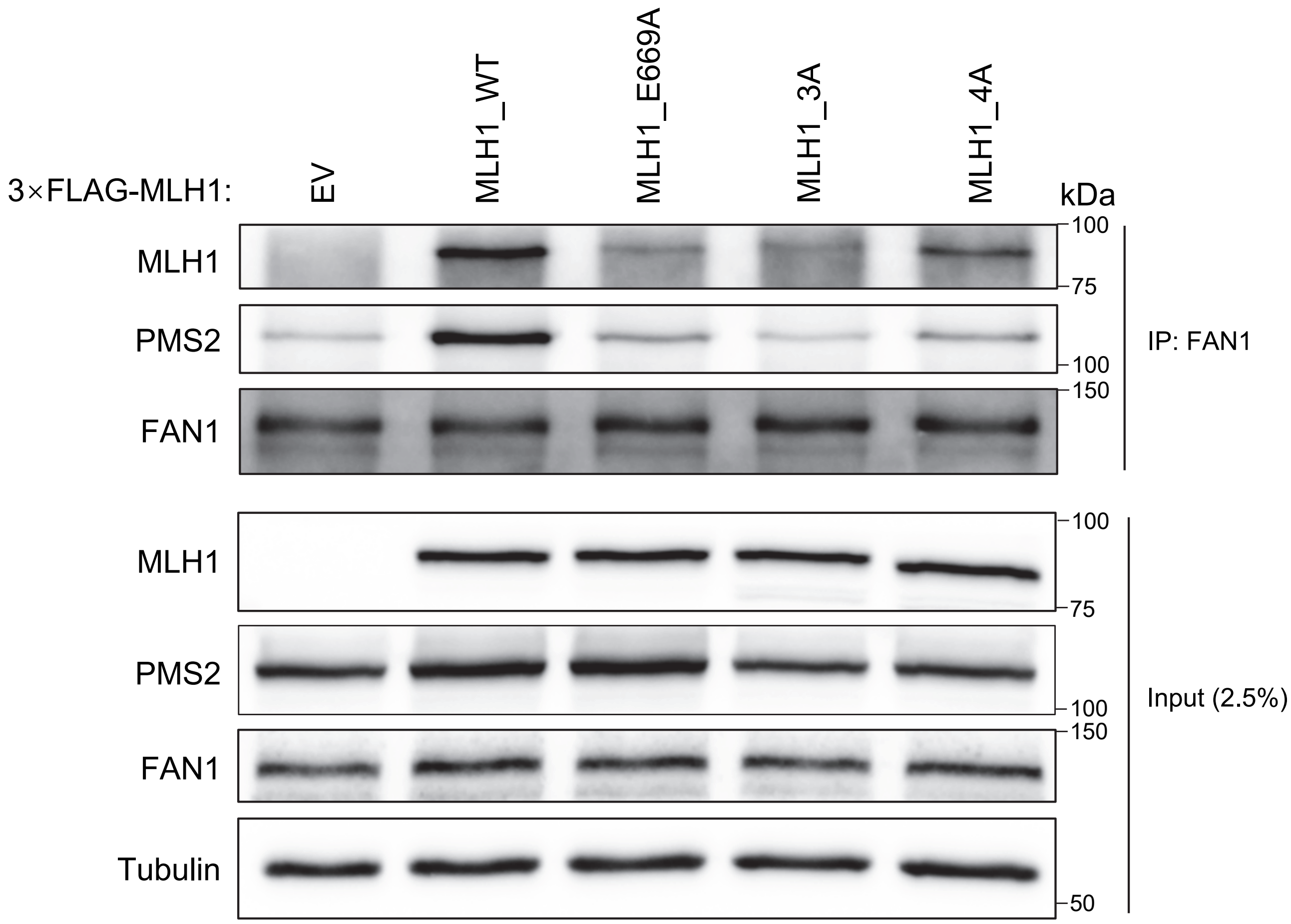

Supplementary Fig. 7 Mutation of essential MLH1 interaction residues impairs FAN1 binding. Immunoblots of FAN1 co-immunoprecipitates of lysates from HEK293T transfected with either 3×FLAG-EV (empty vector) or the indicated 3×FLAG-MLH1 constructs (transfected at varying amounts to balance expression), along with PMS2. MLH1_3A: L585A/L636A/L653A; MLH1_4A: L574A/L639A/V647A/L650A. Data shown are representative of three independent experiments with similar results. Source data are provided as a Source Data file.

Supplementary Table 1 Data collection and refinement statistics

Complex	MLH1–FAN1-MIP	MLH1–FAN1-MIM
**PDB code**	9WOX	9WOY
**Data collection**		
Space group	I222	$P22_12_1$
Cell dimensions		
a, *b*, *c* (Å)	84.8, 138.6, 142.5	42.6, 59.6, 248.1
α, *β*, *γ* (◦)	90, 90, 90	90, 90, 90
Resolution (Å)	99.34-3.05 (3.13-3.05)	30.46-2.18 (2.24-2.18)
Measured reflections	206541 (14877)	398548 (17954)
Unique reflections	16365 (1190)	33445 (2233)
R_{merge}	0.041 (0.233)	0.235 (0.450)
$I/\sigma I$	7.3 (1.7)	5.9 (3.4)
$CC_{1/2}$	0.995 (0.931)	0.961 (0.902)
Completeness (%)	99.8 (99.6)	98.1 (90.1)
Redundancy	12.6 (12.5)	11.9 (8.0)
**Refinement**		
Resolution (Å)	36.43-3.05 (3.16-3.05)	30.08-2.28 (2.36-2.28)
R_{work}/R_{free} (%)	23.1/28.9	23.4/27.4
No. of atoms/average *B*-factors ($Å^2$)	3971/105.9	4297/61.1
Protein	3833/106.0	2002/51.5
Ligand	138/119.7	1885/69.7
Water		216/59.5
Root mean square deviation		
Bond lengths (Å)	0.030	0.010
Bond angles (°)	1.7	1.4
Ramachandran plot residues (%)		
Favored	94.9	96.3
Additional allowed	5.1	3.7
Disallowed	0.0	0.0

Values in parentheses are for the highest resolution shell.

Supplementary Table 2 Primers used in this study

Primer name	sequence（5′ to 3′）
MLH1_495_F	TTGTATTTCCAGGGCACCCCCCGGAGAAGGATC
MLH1_486_F	TTGTATTTCCAGGGCTCCCGAAAGGAAATGACTGC
MLH1_751_R	AGTCTGCAGGGTACCTCATTTGTATAGATCAGGCAGG
FAN1_118_F	TTGTATTTCCAGGGCCTGGTGCCGCGCGGCAGC
FAN1_177_R	AGTCTGCAGGGTACCTCATTACGCAAATTCTTCATCTTTATC
MLH1_495_F (for Duet construct)	TAAGAAGGAGATATACATATGACCCCCCGGAGAAGGATCATT
MLH1_751_R (for Duet construct)	TTTACCAGACTCGAGTCATTTGTATAGATCAGGCAGGTTAG
FAN1_118_F (for Duet construct)	TTGTATTTCCAGGGCCTGGTGCCGCGCGGCAGCAAACG
FAN1_177_R (for Duet construct)	CAAGCTTCGTCATCATTACGCAAATTCTTCATCTTTATC
MLH1_L574A_F	TGGTGTTGCCAGGTTATCGGAGCCAGCACCGC
MLH1_L574A_R	ATAACCTGGCAACACCAAAATTGGCAAAATCAT
MLH1_S577A_F	TTCTCAGGTTAGCGGAGCCAGCACCGCTCTTTG
MLH1_S577A_R	CTCCGCTAACCTGAGAACACCAAAATTGGCAA
MLH1_L585A_F	TCTTTGACGCTGCCATGCTTGCCTTAGATAGTCC
MLH1_L585A_R	CATGGCAGCGTCAAAGAGCGGTGCTGGCTCCG
MLH1_M621A_F	AGGCGCTTGCAGACTATTTCTCTTTGGAAATT
MLH1_M621A_R	ATAGTCTGCAAGCGCCTCAGCCTTCTTCTTCAGAAACTCA
MLH1_D624A_F	GAGATGCTTGCAGCATATTTCTCTTTGGAAATTGATGAGGA
MLH1_D624A_R	TATGCTGCAAGCATCTCAGCCTTCTTCTTCAG
MLH1_Y625A_F	TGCAGACGCTTTCTCTTTGGAAATTGATGAGGAA
MLH1_Y625A_R	AAGAGAAAGCGTCTGCAAGCATCTCAGCCTTC
MLH1_E629A_F	GGCAATTGATGAGGAAGGGAACCTGATTGGAT
MLH1_E629A_R	CTTCCTCATCAATTGCCAAAGAGAAATAGTCTGCAAGC
MLH1_L636A_F	AGGGAACGCGATTGGATTACCCCTTCTGATTGAC
MLH1_L636A_R	ATCCAATCGCGTTCCCTTCCTCATCAATTTCCA
MLH1_L639A_F	TGATTGGAGCTCCCCTTCTGATTGACAACTATGTG
MLH1_L639A_R	AAGGGGAGCTCCAATCAGGTTCCCTTCCTCAT
MLH1_V647A_F	AACTATGCGCCCCCTTTGGAGGGACTGCCTAT
MLH1_V647A_R	AAAGGGGGCGCATAGTTGTCAATCAGAAGGGGTAATCC

MLH1_L650A_F	TATGTGCCCCCTGCGGAGGGACTGCCTATCTTCATTCTT
MLH1_L650A_R	TCCGCAGGGGGCACATAGTTGTCAATCAGAAG
MLH1_L653A_F	GGAGCGCCTATCTTCATTCTTCGACTAGCCAC
MLH1_L653A_R	ATGAAGATAGGCGCTCCCTCCAAAGGGGGCAC
MLH1_E669A_F	GGACGAAGCAAAGGAATGTTTTGAAAGCCTCAGT
MLH1_E669A_R	ATTCCTTTGCTTCGTCCCAATTCACCTCAGTG
MLH1_F673A_F	GGAATGTGCTGAAAGCCTCAGTAAAGAATGCGC
MLH1_F673A_R	GGCTTTCAGCACATTCCTTTTCTTCGTCCCAA
MLH1_V647A/L650A_F	AACTATGCGCCCCCTGCGGAGGGACTGCCTATCTTCATTCTT
MLH1_V647A/L650A_R	CGCAGGGGGCGCATAGTTGTCAATCAGAAGGGGTAATCC
MLH1_L639A/V647A/L650A_F	GCTCCCCTTCTGATTGACAACTATGCGCCCCCTGCGGAGGGACTGCCTATCTTCATTCTT
MLH1_L639A/V647A/L650A_R	TCCGCAGGGGGCGCATAGTTGTCAATCAGAAGGGGAGCTCCAATCAGGTTCCCTTCCTC

The primer sequences for *FAN1* are based on synthetic codon optimized FAN1 coding sequence.
