# Supplementary material for: Structural insights into the MLH1–FAN1 interaction reveal an uncharacterized binding interface on MLH1
Source: Nat Commun. 2026 Jul 6;17:8367. doi: 10.1038/s41467-026-74991-0 (PMC13473659; doi:10.1038/s41467-026-74991-0)
Supplement: Supplementary file 2 [file 41467_2026_74991_MOESM2_ESM.pdf]

nature portfolio

Corresponding author(s): Yanli Liu

Last updated by author(s): Jun 13, 2026

Reporting Summary

Nature Portfolio wishes to improve the reproducibility of the work that we publish. This form provides structure for consistency and transparency in reporting. For further information on Nature Portfolio policies, see our Editorial Policies and the Editorial Policy Checklist.

Statistics

For all statistical analyses, confirm that the following items are present in the figure legend, table legend, main text, or Methods section.

n/a	Confirmed	
x	☐	The exact sample size (n) for each experimental group/condition, given as a discrete number and unit of measurement
x	☐	A statement on whether measurements were taken from distinct samples or whether the same sample was measured repeatedly
x	☐	The statistical test(s) used AND whether they are one- or two-sided<br>*Only common tests should be described solely by name; describe more complex techniques in the Methods section.*
x	☐	A description of all covariates tested
x	☐	A description of any assumptions or corrections, such as tests of normality and adjustment for multiple comparisons
x	☐	A full description of the statistical parameters including central tendency (e.g. means) or other basic estimates (e.g. regression coefficient) AND variation (e.g. standard deviation) or associated estimates of uncertainty (e.g. confidence intervals)
x	☐	For null hypothesis testing, the test statistic (e.g. F, t, r) with confidence intervals, effect sizes, degrees of freedom and P value noted<br>*Give P values as exact values whenever suitable.*
x	☐	For Bayesian analysis, information on the choice of priors and Markov chain Monte Carlo settings
x	☐	For hierarchical and complex designs, identification of the appropriate level for tests and full reporting of outcomes
x	☐	Estimates of effect sizes (e.g. Cohen's d, Pearson's r), indicating how they were calculated

Our web collection on statistics for biologists contains articles on many of the points above.

Software and code

Policy information about availability of computer code

Data collection	The diffraction data of the crystals were collected at beamline BL10U2 of the Shanghai Synchrotron Radiation Facility (SSRF) at 100 K. The isothermal titration calorimetry (ITC) assays were performed utilizing an iTC-200 (MicroCal, Inc.). The biolayer interferometry (BLI) assays were conducted using an Octet-R2 system (Sartorius) and the raw kinetic data were collected using Data Acquisition software (Octet BLI Discovery 13.0).
Data analysis	Diffraction images were processed using autoPROC/AIMLESS. The structures were solved by molecular replacement with PHASER using the crystal structure of MLH1-CTD (PDB: 3RBN) as the search model. For both crystal structures, interactive model was built with COOT, followed by initial restrained refinement with REFMAC. The model was then automatically rebuilt with ARP/wARP, further refinement with PHENIX and REFMAC. Restrained refinement and validation were performed with MOLPROBITY. The isothermal titration calorimetry (ITC) data were analyzed using the single-site binding model after subtraction of the control heat signals within the Origin software package (MicroCal, Inc.). The biolayer interferometry (BLI) data of association/dissociation rate constants (kon/koff) were generated with the reference subtraction method using Data Analysis software (Octet Analysis Studio 13.0), and affinities (KD) were calculated accordingly.

For manuscripts utilizing custom algorithms or software that are central to the research but not yet described in published literature, software must be made available to editors and reviewers. We strongly encourage code deposition in a community repository (e.g. GitHub). See the Nature Portfolio guidelines for submitting code & software for further information.

Data

Policy information about availability of data

All manuscripts must include a data availability statement. This statement should provide the following information, where applicable:

- Accession codes, unique identifiers, or web links for publicly available datasets
- A description of any restrictions on data availability
- For clinical datasets or third party data, please ensure that the statement adheres to our policy

The atomic coordinates and structure factors have been deposited in the Protein Data Bank (PDB) with accession codes 9WOX (https://www.rcsb.org/structure/unreleased/9WOX, MLH1-CTD–FAN1-MIP) and 9WOY (https://www.rcsb.org/structure/unreleased/9WOY, MLH1-CTD–FAN1-MIM), respectively. The structural data used in this study are available in the Protein Data Bank (PDB) under accession codes 3RBN (https://www.rcsb.org/structure/3RBN, MLH1-CTD apo structure), 4FMN (https://www.rcsb.org/structure/4FMN, ScMlh1-CTD–ScPms1-CTD–ScNtg2-MIP complex), 4FMO (https://www.rcsb.org/structure/4FMO, ScMlh1-CTD–ScPms1-CTD–ScExo1-MIP complex), 1X9Z (https://www.rcsb.org/structure/1X9Z, EcMutL-CTD), 6RMN (https://www.rcsb.org/structure/6RMN, ScMlh1-CTD–ScMlh3-CTD complex), respectively.

Research involving human participants, their data, or biological material

Policy information about studies with human participants or human data. See also policy information about sex, gender (identity/presentation), and sexual orientation and race, ethnicity and racism.

Reporting on sex and gender	n/a
Reporting on race, ethnicity, or other socially relevant groupings	n/a
Population characteristics	n/a
Recruitment	n/a
Ethics oversight	n/a

Note that full information on the approval of the study protocol must also be provided in the manuscript.

Field-specific reporting

Please select the one below that is the best fit for your research. If you are not sure, read the appropriate sections before making your selection.

- [x] Life sciences
- [ ] Behavioural & social sciences
- [ ] Ecological, evolutionary & environmental sciences

For a reference copy of the document with all sections, see nature.com/documents/nr-reporting-summary-flat.pdf

Life sciences study design

All studies must disclose on these points even when the disclosure is negative.

Sample size	Based pilot studies, experiments were performed at 2-3 times to verify the reproducibility of the experimental findings. The exact sample size of the associated experiments was described in the Method and Figure legends.
Data exclusions	No data were excluded.
Replication	Each assay was performed at 2-3 times. All the attempts at replication were successful.
Randomization	Randomization was not considered within the experimental samples tested in this study, since the cell line used was generally identical.
Blinding	Blinding was not considered within the experimental samples tested in this study, since data generated in this study was not practical and no subjective results were gathered.

Reporting for specific materials, systems and methods

We require information from authors about some types of materials, experimental systems and methods used in many studies. Here, indicate whether each material, system or method listed is relevant to your study. If you are not sure if a list item applies to your research, read the appropriate section before selecting a response.

Materials & experimental systems

n/a	Involved in the study	
☐	☒	Antibodies
☐	☒	Eukaryotic cell lines
☒	☐	Palaeontology and archaeology
☒	☐	Animals and other organisms
☒	☐	Clinical data
☒	☐	Dual use research of concern
☒	☐	Plants

Methods

n/a	Involved in the study	
☒	☐	ChIP-seq
☒	☐	Flow cytometry
☒	☐	MRI-based neuroimaging

Antibodies

Antibodies used

Primary antibodies:
anti-FAN1: RRID: AB_10598016, Proteintech, 17600-1-AP, rabbit polyclonal antibody
anti-MLH1: RRID: AB_2049968, Abcam, ab92312, rabbit monoclonal antibody, clone number EPR3895
anti-PMS2: RRID: AB_10858491, Abcam, ab110638, rabbit monoclonal antibody, clone number EPR3947
anti-GAPDH: RRID: AB_2107445, Millipore, MAB374, mouse monoclonal antibody, clone number 6C5
anti-tubulin: RRID: AB_477593, Sigma-Aldrich, T9026, mouse monoclonal antibody, clone number DM1A
anti-cyclin D1: RRID: AB_2228523, Cell Signaling, 2922, rabbit polyclonal antibody

Secondary antibodies:
horseradish peroxidase-conjugated donkey anti-rabbit secondary antibody: RRID: AB_772206, Cytiva, NA934
horseradish peroxidase-conjugated sheep anti-mouse secondary antibody: RRID: AB_772210, Cytiva, NA931

Validation

All the antibodies used in this study were obtained from commercial companies and have been registered with a unique Research resource identifier (RRID). Validation of these antibodies are accessible on companies' websites or RRID portal and all the antibodies were verified by Relative expression to ensure that the antibody binds to the antigen stated.

Eukaryotic cell lines

Policy information about cell lines and Sex and Gender in Research

Cell line source(s)

HEK293 cells (ATCC, CRL-11268) were obtained from ATCC.

Authentication

The cell line was authenticated by short tandem repeat (STR) profile method.

Mycoplasma contamination

The cell line was tested negative for mycoplasma contamination by PCR.

Commonly misidentified lines
(See ICLAC register)

No commonly misidentified line was used in this study.

Plants

Seed stocks

n/a

Novel plant genotypes

n/a

Authentication

n/a
