# Supplementary material for: Structural insights into the MLH1–FAN1 interaction reveal an uncharacterized binding interface on MLH1
Source: Nat Commun. 2026 Jul 6;17:8367. doi: 10.1038/s41467-026-74991-0 (PMC13473659; doi:10.1038/s41467-026-74991-0)
Supplement: Supplementary file 3 [file 41467_2026_74991_MOESM3_ESM.pdf]

nature portfolio

Peer Review File

Structural insights into the MLH1–FAN1 interaction reveal an uncharacterized binding interface on MLH1

Corresponding Author: Dr Yanli Liu

This file contains all reviewer reports in order by version, followed by all author rebuttals in order by version.

Attachments originally included by the reviewers as part of their assessment can be found at the end of this file.

Version 0:

Reviewer comments:

Reviewer #1

(Remarks to the Author)
The manuscript of Chen and co-workers describes the first high resolution structure of MIB-FAN1 and MIM-FAN1 peptides and their interaction with the MLH1-C-terminal peptide. The MLH1-FAN1 complex has an important modulatory impact the CAG tract size, which underlies the age of HD onset. Thus, the work is timely and relevant.

The C-terminal domain of MLH1 recognizes a canonical MIP motif (residues 124-131) and a downstream at MIM motif (residues 152-163) within its N-terminus of FAN1. Using a combination of binding affinity measurements, crystal structures and mutagenesis, the authors define the structural features of these associations using purified peptides containing the MIP and MIM motifs of FAN 1 and the MLH1-C-terminal peptide. The ITC data confirm that both the FAN-1-MIP and FAN-1-MIM bind to MLH1-C-terminal peptide, but MIP and MIM motifs have distinct features. The FAN1-MIP-MLH1-C-terminal peptide complex forms hydrogen bond (S-126) but is primarily stabilized through hydrophobic stacking at a previously identified “S2” site (the SxYF motif). The FAN-1 MIM binds to the MLH1-C-terminal peptide at a third site (“S3”), which overlaps with binding site for MSH3-MIP. Interestingly, the MSH3-MIP binds to MLH1-CTD with much weaker binding affinity compared to FAN1-MIP, suggesting that the two peptides can compete and that FAN1 association may dominate. The MLH1-3A mutants impair hydrophobic contact with the C-terminal region of FAN1-MIM and completely abolishes FAN1 binding.

Overall, this is good work. Its major strength is creation of a structural framework for MLH1-FAN1 complex and its impacts on expansion biology. Experimentally, the authors nicely show that the destabilizing outcomes of mutagenesis predicted by the structural features are supported by the ITC results, providing some functional validation of the structure. The competition of the FAN1-MIM and the MSH3-MIP provides insight into a potential mechanism by which FAN1 interferes with the interaction of MSH3–MLH1 to block expansion (validated by IP reaction). Although disruption of protein-protein contacts is not always functionally productive, identifying the stabilizing residues provides possible targeting sites for design of ligands that might inhibit expansion.

There are some weaknesses. Although the authors provide distinct structures for the MIP and MIM peptide interactions independently, they lack data in the longer peptide (MIM +MIP) which binds with highest affinity. Are the S2 and S3 interaction sites maintained when both are present in a single peptide? Is the longer peptide a competitor for MSH3-MIP? It would enhance the paper to include the longer peptide in the structural analysis. The final purified peptides used in each analysis should be shown-free from Ni++ column background. It is also worrisome that the two binding affinity techniques do not agree, but both are used to substantiate structural results in the paper—which one is correct? Does it change the conclusions if the other is used? The authors should comment. Given that expansion can be followed, it would be particularly nice if the FAN-1-MIM peptide could be directly tested as a blocker of expansion in a cell system (and active expansion restored by mutants). Finally, polymorphisms in FAN1 have distinct effects on whether onset of HD is delayed. Are any such polymorphisms contained in the peptides used? Can the authors comment? If so, a Table comparing the positions of polymorphisms, their functional effects with respect to the structural predictions would provide rich information that would enhance the significance of the structure if a correlation can be made.

Reviewer #2

(Remarks to the Author)
This paper provides direct evidence of interaction between MLH1 CTD and FAN1 MIP and MIM regions. Robust interactions were observed in vitro in quantitative binding assays (ITC and BLI). X-ray crystallography was used to map the interaction sites and the main physicochemical interactions at the residue level. Both the conserved FAN1 MIP engagement site in the CTD of MLH1 was mapped, and a novel binding site (S3) for engagement of the FAN1 MIM were identified. These in vitro biophysical observations were validated in a cellular context using co-immunoprecipitation, with interaction being disrupted upon mutation of key MLH1 binding sites.

This is a compelling body of work that probes a system using multiple orthogonal techniques. ITC and BLI were used to probe the interaction in both surface-based and in solution contexts. KD values are provided, giving quantitative understanding of the interactions between different binding partners. Extensive work was also done to corroborate biophysical analyses with co-immunoprecipitation assays that provide insights into interactions between MLH1 and FAN1 in the cellular context.

Some points that i would like the authors to clarify are

"While ITC detected binding for the shorter FAN1-MIM (residues 152–163) peptide, no detectable binding was observed for the longer FAN1-MIM (residues 145–163) peptide (data not shown)" Can they expand on this? it is counterintuitive as the longer peptide contains the same residues as the shorter one. Also i believe later they mention that binding is stronger?

For binding affinities both ITC and BLI are used which report fairly different values, I would like the authors to provide an explanation for this but also which of the two should the readers 'trust' more and why.

Why does the MLH1_3A mutant abolish FAN1 coIP, and the MLH1_4A mutant not? Seems curious and just wondering that would be the case.

In Figure 5 What happens to FAN1 in the MLH1_E669A lane? Is it degradation?

Reviewer #3

(Remarks to the Author)
This manuscript reports the crystal structures of the human MLH1 C-terminal domain (MLH1-CTD) in complex with either the FAN1 MIP or MIM peptide. MLH1, a central component of the mismatch repair pathway, also plays a key role in regulating short nucleotide repeat instability in humans. When MLH1 associates with MutSβ, it promotes repeat expansion; in contrast, when MLH1 engages the nuclease FAN1, the complex suppresses expansions and instead promotes repeat contractions. The authors have further validated the MLH1–FAN1 interacting interfaces through mutagenesis, isothermal titration calorimetry (ITC), bio-layer interferometry (BLI), and cellular coimmunoprecipitation assays. Using classical biochemical approaches combined with crystallography, the study convincingly delineates how MLH1 and FAN1 interact to mediate their function in limiting the expansion of short nucleotide repeats.
It would strengthen the manuscript to examine whether the newly identified s3 site in human MLH1 affects repeat stability in cellular models. Nevertheless, the identification of a previously uncharacterized MLH1 binding surface (site s3), together with the experimental evidence and clear presentation, supports publication in Nature Communications. However, explanation of some experiment design and revision to address the points outlined below would enhance the clarity and impact of the work.
1. Mutation design of 3A and 4A in MLH1-CTD
The MLH1_3A and 4A mutations were designed to disrupt the MLH1–FAN1 (MIM) interface. When the 3A and 4A mutations are displayed on the 2.2 Å crystal structure of MLH1-CTD (PDB: 3RBN), one residue in each patch belongs to the hydrophobic core (L636A in MLH1_3A and L639A in MLH1_4A) (see left). Mutating them would destabilize the MLH1-CTD structure, which may explain the formation of inclusion bodies of these mutant proteins when over-expressed. The authors need to explain the nature of these mutations and the effects of a destabilized MLH1-CTD on its reduced binding to FAN1.
Surprisingly, L576, which is exposed and covers L636 and L639, is not mutated to Ala or charged sidechain to verify the importance of site s3. A diagram showing the locations of the mutated residues in MLH1-3A and 4A and their solvent accessibility is needed.

2. Potential influence of MutL dimerization on MLH1–FAN1 interactions
MLH1-CTD forms constitutive heterodimers with its partners PMS2, PMS1, or MLH3. The MutL dimerization interface typically extends beyond the s1 site and involves the extreme C-terminus. Could dimerization within MutL complexes influence, either positively or negatively, the interaction between human MLH1 and FAN1? Clarification or discussion of this possibility would be helpful.
3. Placement and visualization of MLH1 binding-site summary
The combined summary of the three MLH1-CTD binding sites appears only in Fig. 8e. Because these sites are central to the experimental design and conclusions, presenting this figure panel earlier in the manuscript would improve readability. The authors may consider illustrating their spatial relationships from more than one orientation, ideally with electrostatic surface potentials, to better convey the nature of the interactions.
4. Inclusion of peptide electron densities
Given the moderate resolution of the crystal structures, including electron density maps for the MIP and MIM peptides in Fig. 2a and Fig. 4a would convince readers of the modeled interactions.
5. Effects of FAN1 on mismatch repair
Interactions between MutSβ and MutL complexes are essential for mismatch repair. The manuscript reports substantially weaker binding of MLH1-CTD to the MSH3-MIP peptide compared to FAN1-MIP. What are the implications of such weak MLH1–MSH3 interactions? What functional consequences would be when the MLH1 s2 site (the MIP-binding region) is mutated with respect to mismatch repair efficiency and repeat stability? Further discussion could strengthen the mechanistic interpretation.

Version 1:

Reviewer comments:

Reviewer #1

(Remarks to the Author)
The manuscript of Chen and co-workers describes the first high resolution structure of MIB-FAN1 and MIM-FAN1 peptides and their interaction with the MLH1-C-terminal peptide. The MLH1-FAN1 complex has an important modulatory impact the CAG tract size, which underlies the age of HD onset. Thus, the work is timely and relevant.

Th authors have addressed my questions. The thermal melting issues were well explained and makes sense, the structural results were also validated using IP reaction. The S3 site is a useful discovery. Overall, this work creates a nice beginning of a structural framework for MLH1-FAN1 complex and its impacts on expansion biology.

Reviewer #3

(Remarks to the Author)
The authors have addressed all my questions. The revised manuscript is suitable for publication.

Open Access This Peer Review File is licensed under a Creative Commons Attribution 4.0 International License, which permits use, sharing, adaptation, distribution and reproduction in any medium or format, as long as you give appropriate credit to the original author(s) and the source, provide a link to the Creative Commons license, and indicate if changes were made.
In cases where reviewers are anonymous, credit should be given to 'Anonymous Referee' and the source.
The images or other third party material in this Peer Review File are included in the article's Creative Commons license, unless indicated otherwise in a credit line to the material. If material is not included in the article's Creative Commons license and your intended use is not permitted by statutory regulation or exceeds the permitted use, you will need to obtain permission directly from the copyright holder.
To view a copy of this license, visit https://creativecommons.org/licenses/by/4.0/

General Response: We thank all the reviewers for their insightful comments and constructive suggestions, which have helped us significantly improve the manuscript. We have conducted additional experiments and analyses in response to the points raised, and have revised the text and figures accordingly. Our point-by-point responses are detailed below.

Reviewer #1 (Remarks to the Author)

The manuscript of Chen and co-workers describes the first high resolution structure of MIB-FAN1 and MIM-FAN1 peptides and their interaction with the MLH1-C-terminal peptide. The MLH1-FAN1 complex has an important modulatory impact the CAG tract size, which underlies the age of HD onset. Thus, the work is timely and relevant.

The C-terminal domain of MLH1 recognizes a canonical MIP motif (residues 124-131) and a downstream at MIM motif (residues 152-163) within its N-terminus of FAN1. Using a combination of binding affinity measurements, crystal structures and mutagenesis, the authors define the structural features of these associations using purified peptides containing the MIP and MIM motifs of FAN 1 and the MLH1-C-terminal peptide. The ITC data confirm that both the FAN-1-MIP and FAN-1-MIM bind to MLH1-C-terminal peptide, but MIP and MIM motifs have distinct features. The FAN1-MIP-MLH1-C-terminal peptide complex forms hydrogen bond (S-126) but is primarily stabilized through hydrophobic stacking at a previously identified “S2” site (the SxYF motif). The FAN-1 MIM binds to the MLH1-C-terminal peptide at a third site (“S3”), which overlaps with binding site for MSH3-MIP. Interestingly, the MSH3-MIP binds to MLH1-CTD with much weaker binding affinity compared to FAN1-MIP, suggesting that the two peptides can compete and that FAN1 association may dominate. The MLH1-3A mutants impair hydrophobic contact with the C-terminal region of FAN1-MIM and completely abolishes FAN1 binding.

Overall, this is good work. Its major strength is creation of a structural framework for MLH1-FAN1 complex and its impacts on expansion biology. Experimentally, the authors nicely show that the destabilizing outcomes of mutagenesis predicted by the structural features are supported by the ITC results, providing some functional validation of the structure. The competition of the FAN1-MIM and the MSH3-MIP provides insight into a potential mechanism by which FAN1 interferes with the interaction of MSH3–MLH1 to block expansion (validated by IP reaction). Although disruption of protein-protein contacts is not always functionally productive, identifying the stabilizing residues provides possible targeting sites for design of ligands that might inhibit expansion.

Response: We appreciate the reviewer’s positive assessment of our work.

1. There are some weaknesses. Although the authors provide distinct structures for the MIP and MIM peptide interactions independently, they lack data in the longer peptide (MIM +MIP) which binds with highest affinity. Are the S2 and S3 interaction sites maintained when both are present in a single peptide? It would enhance the paper to include the longer peptide in the structural analysis.

   Response: We agree that a structure of the full complex would be highly informative. Despite extensive efforts, including co-crystallization of MLH1-CTD with the FAN1-MIP+MIM peptide (residues 124–163) and co-expression/purification strategies, we were unable to obtain crystals of this complex. We attribute this primarily to the presence of a flexible loop connecting the MIP and MIM motifs, as suggested by our AlphaFold Multimer model (now included as Fig. 8d). This model strongly supports the idea that both sites are engaged simultaneously, showing the binding of the FAN1-MIP+MIM fragment to the S2 and S3 sites of MLH1-CTD via its MIP-box and MIM motif, respectively (Fig. 8d). We have added this point in the Discussion in the revised manuscript (end of section "A novel binding site on MLH1-CTD engages the FAN1-MIM motif").

2. Is the longer peptide a competitor for MSH3-MIP?

   Response: Yes. Since both FAN1-MIP and MSH3-MIP bind to the same S2 site on MLH1-CTD, competition is expected. Our competitive ITC data confirm that FAN1-MIP competes with MSH3-MIP (Supplementary Fig. 3a). We have now performed the same experiment with the longer FAN1-MIP+MIM peptide and observed an identical competitive effect (new data provided below), further supporting the mechanistic relevance of this competition.

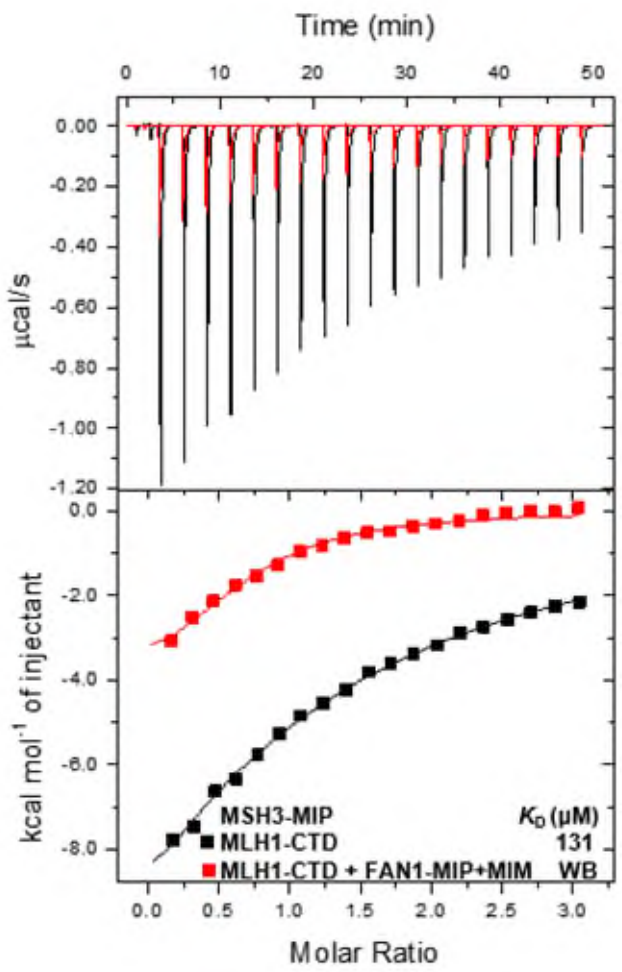

3. The final purified peptides used in each analysis should be shown-free from Ni++ column background.

   Response: All the proteins used in the manuscript were further purified by gel-

filtration column after Ni++ column. All the peptides used in the manuscript were synthesized by the company without purification by Ni++ column.

4. It is also worrisome that the two binding affinity techniques do not agree, but both are used to substantiate structural results in the paper—which one is correct? Does it change the conclusions if the other is used? The authors should comment.

   Response: We acknowledge the observed differences in absolute affinity values between ITC and BLI. This is not uncommon, as each technique operates on different physical principles: ITC measures enthalpy changes in solution, while BLI measures binding via changes in optical interference at a sensor surface (Zhou et al, Pharmacol Ther. 2026, 277:108947). Potential factors contributing to the discrepancy include the immobilization chemistry in BLI or different buffer conditions. Although the absolute affinity values varied between techniques, each method provided self-consistent relative comparisons. For instance, both techniques agreed that FAN1-MIP and the core FAN1-MIM motif bind with comparable micromolar-range affinity, and BLI further established that the extended MIM peptide binds with much higher affinity. So, the conclusions drawn within each self-consistent dataset are robust. The convergence of orthogonal methods strengthens our overall mechanistic interpretation. We have added this discussion to the revised manuscript (end of section "MLH1-CTD binds to FAN1-MIP and FAN1-MIM peptides with comparable affinities").

5. Given that expansion can be followed, it would be particularly nice if the FAN-1-MIM peptide could be directly tested as a blocker of expansion in a cell system (and active expansion restored by mutants).

   Response: We agree that this would be an interesting experiment. However, direct delivery of synthetic peptides into cells for a long-term repeat instability assay is technically challenging due to issues with cellular penetration, stability, and sustained effective concentration.

6. Finally, polymorphisms in FAN1 have distinct effects on whether onset of HD is delayed. Are any such polymorphisms contained in the peptides used? Can the authors comment? If so, a Table comparing the positions of polymorphisms, their functional effects with respect to the structural predictions would provide rich information that would enhance the significance of the structure if a correlation can be made.

   Response: We thank the reviewer for this valuable suggestion. Among the disease-associated FAN1 variants, R145H falls within the FAN1-MIM peptide (residues 145–163) used in our structural studies. Our complex structure reveals that FAN1-R145 forms hydrogen bond with MLH1-E629 and fits optimally with the negatively charged surface of MLH1. The R145H mutation, which replaces a

positively charged arginine with a histidine (carrying less positive charge at physiological pH), is predicted to destabilize this interaction by reducing electrostatic complementarity, a finding supported by BLI assays (new Supplementary Fig. 4). This provides a direct structural rationale for the observed correlation between the R145H variant and an earlier, more severe HD phenotype. We have incorporated this analysis into the revised manuscript (in the section "FAN1-MIM (residues 145–163) peptide binds to a new binding site of MLH1-CTD"). While a comprehensive table of all polymorphisms is beyond the scope of this structural study, the mechanistic insight gained for R145H strongly underscores the functional significance of the interface we have characterized.

Reviewer #2 (Remarks to the Author)

This paper provides direct evidence of interaction between MLH1 CTD and FAN1 MIP and MIM regions. Robust interactions were observed in vitro in quantitative binding assays (ITC and BLI). X-ray crystallography was used to map the interaction sites and the main physicochemical interactions at the residue level. Both the conserved FAN1 MIP engagement site in the CTD of MLH1 was mapped, and a novel binding site (S3) for engagement of the FAN1 MIM were identified. These in vitro biophysical observations were validated in a cellular context using co-immunoprecipitation, with interaction being disrupted upon mutation of key MLH1 binding sites.

This is a compelling body of work that probes a system using multiple orthogonal techniques. ITC and BLI were used to probe the interaction in both surface-based and in solution contexts. KD values are provided, giving quantitative understanding of the interactions between different binding partners. Extensive work was also done to corroborate biophysical analyses with co-immunoprecipitation assays that provide insights into interactions between MLH1 and FAN1 in the cellular context.

Response: We appreciate the reviewer's positive assessment of our work.

Some points that i would like the authors to clarify are

1. "While ITC detected binding for the shorter FAN1-MIM (residues 152–163) peptide, no detectable binding was observed for the longer FAN1-MIM (residues 145–163) peptide (data not shown)" Can they expand on this? it is counterintuitive as the longer peptide contains the same residues as the shorter one. Also i believe later they mention that binding is stronger?

   Response: We appreciate the reviewer's question regarding this initially counterintuitive result. The explanation lies in the distinct binding kinetics of the

two peptides, which are differentially detected by ITC and BLI. Our BLI data provide the key kinetic parameters (Fig. 1c, d, f):

- The shorter FAN1-MIM peptide (residues 152–163) exhibits an association rate (k_{on}) of (6.33 ± 0.34) × 10^4 $M^{-1}s^{-1}$ and a dissociation rate (k_{off}) of 2.09 ± 0.11 s^{-1}, which is compatible with ITC measurement.
- The longer FAN1-MIM peptide (residues 145–163) displays a comparable on-rate of (3.35 ± 0.02) × 10^4 $M^{-1}s^{-1}$, but a dramatically slower off-rate of (1.95 ± 0.02) × 10^{-3} s^{-1}, nearly three orders of magnitude slower than that of the shorter peptide.

The extremely slow dissociation of the longer peptide likely prevented the system from reaching binding equilibrium within the ITC experimental timeframe, explaining the lack of a detectable signal. BLI is particularly suited for characterizing interactions with very slow dissociation rates. Crucially, the BLI data confirm that the longer peptide binds with substantially higher affinity (K_D 0.06 μM *vs*. 33 μM for the shorter peptide), demonstrating that the upstream segment (residues 145–151) critically stabilizes the complex. This is fully consistent with our crystal structure, which reveals that this segment forms additional specific contacts, such as the hydrogen bonds between the main chains of FAN1 residues S146, K148, and I150, as well as the side chain of FAN1-R145, and the main chains of MLH1 residues V573, R575, and S577, together with the side chains of MLH1-S577 and E629, and the hydrophobic interaction between FAN1-I150 and MLH1 residues L574, L639, V647, and L650 (Fig. 4d). We have incorporated this analysis into the revised manuscript (end of section "MLH1-CTD binds to FAN1-MIP and FAN1-MIM peptides with comparable affinities").

2. For binding affinities both ITC and BLI are used which report fairly different values, I would like the authors to provide an explanation for this but also which of the two should the readers 'trust' more and why.

   Response: We appreciate the reviewer's attention to the quantitative details of our binding data. The observed differences in absolute affinity values between ITC and BLI are not uncommon in biophysical studies, as each technique measures binding through fundamentally different physical principles: ITC detects enthalpy changes in solution, whereas BLI monitors changes in optical interference at an immobilized sensor surface (Zhou et al., Pharmacol. Ther. 2026, 277:108947). Discrepancies can arise from several factors, including the immobilization chemistry required for BLI (which may subtly alter protein conformation or accessibility) and differences in buffer conditions or experimental setup.

   Regarding which method readers should "trust" more, it is important to clarify that both are highly validated techniques, and the choice often depends on the scientific

question. ITC is frequently regarded as the gold standard for determining solution-phase thermodynamic parameters (ΔH, ΔS) without the need for labeling or immobilization. However, BLI excels in providing real-time kinetic information (k_{on}, k_{off}) and is invaluable for studying interactions with very slow off-rates, as we encountered. Crucially, the key conclusions of our study do not rely on the absolute K_D value from a single method but are drawn from the consistent trends within each orthogonal dataset. Both techniques independently and unequivocally confirm that FAN1-MIP and FAN1-MIM bind to MLH1-CTD, and key interfacial mutations disrupt binding. The convergence of evidence from these complementary approaches strengthens, rather than weakens, the mechanistic interpretation of our structural work. We have added a discussion of these points to the revised manuscript (end of section "MLH1-CTD binds to FAN1-MIP and FAN1-MIM peptides with comparable affinities").

3. Why does the MLH1_3A mutant abolish FAN1 coIP, and the MLH1_4A mutant not? Seems curious and just wondering that would be the case.

   Response: This difference highlights the non-equivalent roles of the two hydrophobic interfaces. MLH1_4A disrupts the cage for FAN1-I150, while MLH1_3A targets the stacking interface for the FAN1-MIM C-terminus (L152, L155, L159). The more severe effect of the 3A mutant in cellular co-IP suggests that the C-terminal hydrophobic stack is critically important for maintaining the interaction in a cellular context, possibly due to avidity effects or the cellular environment. This is consistent with prior work (Porro et al., Sci Adv, 2021, 7, eabf7906) showing that the FAN1 double mutant L155A/L159A fully disrupts the interaction.

4. In Figure 5 What happens to FAN1 in the MLH1_E669A lane? Is it degradation?

   Response: The reviewer is vigilant in noting the double bands in the FAN1 blot for the MLH1_E669A sample (in Fig. 6, as correctly identified). This is not due to FAN1 degradation. The lower band results from signal bleed-through from the strongly overexpressed PMS2 protein, as indicated by the asterisk in the figure legend. In the MLH1_WT lane, the robust FAN1 signal merges with this faint bleed-through, appearing as a single thicker band. In the MLH1_3A and MLH1_4A lanes, the lower expression and reduced binding to PMS2 result in no visible bleed-through. We have replaced the Fig. 6a with a repeated co-IP data with short exposure time and less PMS2 bleed-through to prevent any misunderstanding. Furthermore, co-IP assays using an anti-FAN1 antibody corroborates the reduced interactions with MLH1_3A and, to a lesser extent, with MLH1_4A (Supplementary Fig. 6, new data).

Reviewer #3 (Remarks to the Author)

This manuscript reports the crystal structures of the human MLH1 C-terminal domain (MLH1-CTD) in complex with either the FAN1 MIP or MIM peptide. MLH1, a central component of the mismatch repair pathway, also plays a key role in regulating short nucleotide repeat instability in humans. When MLH1 associates with MutSβ, it promotes repeat expansion; in contrast, when MLH1 engages the nuclease FAN1, the complex suppresses expansions and instead promotes repeat contractions. The authors have further validated the MLH1–FAN1 interacting interfaces through mutagenesis, isothermal titration calorimetry (ITC), bio-layer interferometry (BLI), and cellular co-immunoprecipitation assays. Using classical biochemical approaches combined with crystallography, the study convincingly delineates how MLH1 and FAN1 interact to mediate their function in limiting the expansion of short nucleotide repeats.

It would strengthen the manuscript to examine whether the newly identified s3 site in human MLH1 affects repeat stability in cellular models. Nevertheless, the identification of a previously uncharacterized MLH1 binding surface (site s3), together with the experimental evidence and clear presentation, supports publication in Nature Communications. However, explanation of some experiment design and revision to address the points outlined below would enhance the clarity and impact of the work.

Response: We appreciate the reviewer's positive assessment of our work.

1. Mutation design of 3A and 4A in MLH1-CTD

   The MLH1_3A and 4A mutations were designed to disrupt the MLH1–FAN1 (MIM) interface. When the 3A and 4A mutations are displayed on the 2.2 Å crystal structure of MLH1-CTD (PDB: 3RBN), one residue in each patch belongs to the hydrophobic core (L636A in MLH1_3A and L639A in MLH1_4A) (see left).

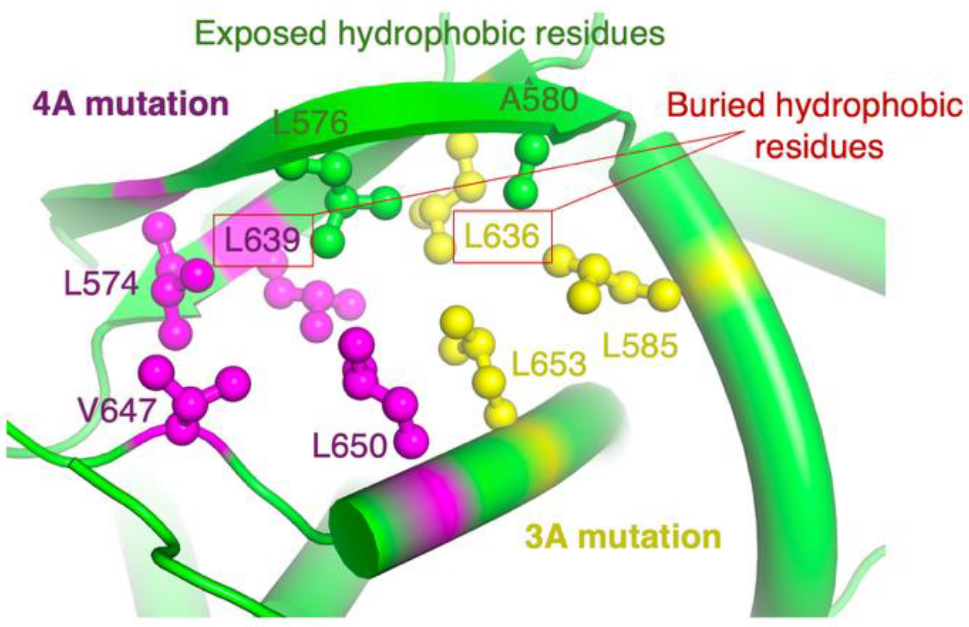

   Mutating them would destabilize the MLH1-CTD structure, which may explain the formation of inclusion bodies of these mutant proteins when over-expressed. The authors need to explain the nature of these mutations and the effects of a destabilized MLH1-CTD on its reduced binding to FAN1. Surprisingly, L576, which is exposed and covers L636 and L639, is not mutated to Ala or charged sidechain to verify the importance of site s3. A diagram showing the locations of the mutated residues in MLH1-3A and 4A and their solvent accessibility is needed.

   Response: We thank the reviewer for this important point. Structural analysis

indeed shows that L636 (in 3A) and L639 (in 4A) are part of the hydrophobic core (new Supplementary Fig. 5a, b). Mutating these core residues, especially in combination, is predicted to destabilize the MLH1-CTD fold, explaining the initial insolubility. To obtain soluble protein for biophysical assays, we used an N-terminal MBP solubility tag. Circular dichroism spectroscopy confirmed the mutants retained a wild-type-like fold (Supplementary Fig. 5c, d), indicating that while the mutations weaken the FAN1 interface, they do not cause global unfolding. We have revised the manuscript to clarify this (in the middle of section "Both the FAN1-MIM motif and its upstream segment are essential for the interaction with MLH1").

Regarding L576A, as suggested, we have now tested it and found it binds to FAN1-MIM with wild-type affinity (new data below), similar to other individual mutations. However, we were unable to purify the multiple point mutant that includes L576A, as the yield was too low.

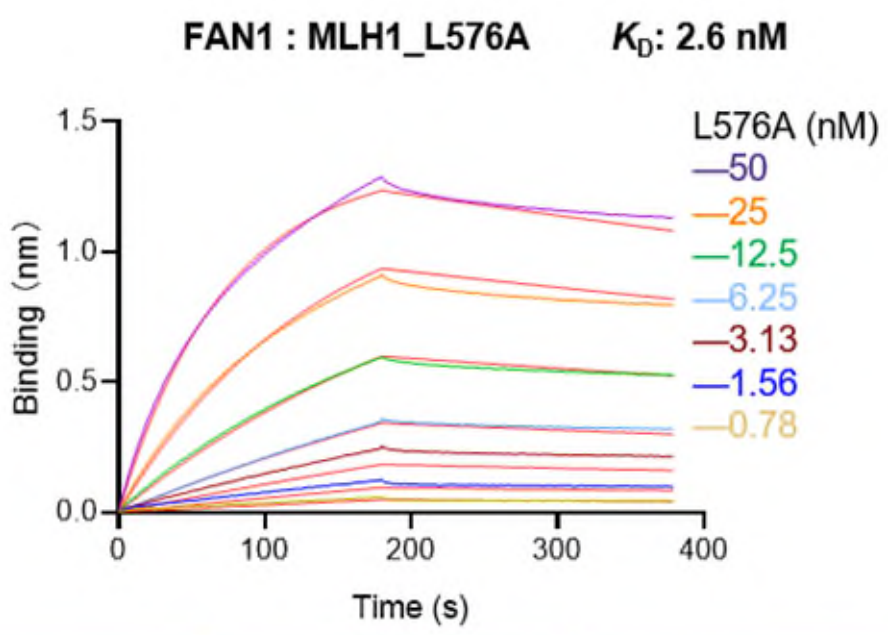

K_D (nM)	k_{on} (10^5 1/Ms)	k_{off} (10^{-4} 1/s)	R^2
2.6 ± 0.02	2.6 ± 0.005	6.7 ± 0.06	0.99

2. Potential influence of MutL dimerization on MLH1–FAN1 interactions
MLH1-CTD forms constitutive heterodimers with its partners PMS2, PMS1, or MLH3. The MutL dimerization interface typically extends beyond the s1 site and involves the extreme C-terminus. Could dimerization within MutL complexes influence, either positively or negatively, the interaction between human MLH1 and FAN1? Clarification or discussion of this possibility would be helpful.
Response: We thank the reviewer for raising this important mechanistic question. Our response addresses two aspects: (1) whether FAN1 binding and MutL dimerization are mutually exclusive, and (2) whether heterodimer formation quantitatively modulates the MLH1–FAN1 interaction.

Regarding the first point, our structural analysis clearly shows that the FAN1-binding sites (S2 for MIP and S3 for MIM) are spatially distinct from the S1 heterodimerization interface. Consistent with this observation, previous studies have demonstrated that MLH1 efficiently co-precipitates PMS2 in cells expressing FAN1 (Goold et al., Cell Rep, 2021, 36, 109649), and that recombinant GST-FAN1

effectively pulls down pre-formed MutLα (MLH1–PMS2) and MutLγ (MLH1–MLH3) complexes (Porro et al., Sci Adv, 2021, 7, eabf7906). These findings indicate that FAN1 binding does not sterically preclude MutL heterodimer formation, nor does dimerization prevent FAN1 engagement.

Regarding the second point—whether dimerization quantitatively influences binding affinity—we acknowledge that this remains an open question. We had initially planned to address this by directly comparing FAN1 binding to MLH1-CTD alone versus the MLH1–PMS2 heterodimer. However, despite multiple attempts, we were unable to obtain purified, well-behaved PMS2-CTD protein suitable for such comparative binding assays. Given the spatial separation between the S2/S3 binding surfaces and the S1 dimerization interface (Fig. 8c, 8e), we consider it unlikely that heterodimer formation induces a conformational change that dramatically alters FAN1 binding. Nonetheless, we agree with the reviewer that a definitive answer would require direct quantitative measurements, and we have added a discussion of this point in the revised manuscript as a future direction (end of section "A novel binding site on MLH1-CTD engages the FAN1-MIM motif").

3. Placement and visualization of MLH1 binding-site summary

   The combined summary of the three MLH1-CTD binding sites appears only in Fig. 8e. Because these sites are central to the experimental design and conclusions, presenting this figure panel earlier in the manuscript would improve readability. The authors may consider illustrating their spatial relationships from more than one orientation, ideally with electrostatic surface potentials, to better convey the nature of the interactions.

   Response: We agree with the reviewer that an early, clear overview of the binding sites is crucial. Accordingly, we have created a new summary panel (Fig. 4f) featuring an electrostatic potential surface representation of MLH1-CTD. This panel clearly delineates the S1 (dimerization), S2 (MIP-binding), and S3 (MIM-binding) sites with black dashed circles, accompanied by enlarged views of each site in both ligand-free and ligand-bound states. The ligand-bound view for the S1 site is modeled using the *Saccharomyces cerevisiae* Mlh3 fragment (PDB: 6RMN). Furthermore, we have moved the corresponding descriptive text forward, adding a new concluding paragraph to the "FAN1-MIM (residues 145–163) peptide binds to a new binding site of MLH1-CTD" section. This revision provides readers with a comprehensive spatial and functional understanding of MLH1-CTD as a multi-functional interaction hub early in the results.

4. Inclusion of peptide electron densities

   Given the moderate resolution of the crystal structures, including electron density

maps for the MIP and MIM peptides in Fig. 2a and Fig. 4a would convince readers of the modeled interactions.

Response: As suggested, we have now included the $2F_o–F_c$ electron density maps for both the FAN1-MIP and FAN1-MIM peptides, contoured at 1σ. These maps are presented in new panels (Fig. 2b and Fig. 4b) and provide clear evidence for the reliability of the modeled peptide conformations in our crystal structures.

5. Effects of FAN1 on mismatch repair

Interactions between MutSβ and MutL complexes are essential for mismatch repair. The manuscript reports substantially weaker binding of MLH1-CTD to the MSH3-MIP peptide compared to FAN1-MIP. What are the implications of such weak MLH1–MSH3 interactions? What functional consequences would be when the MLH1 s2 site (the MIP-binding region) is mutated with respect to mismatch repair efficiency and repeat stability? Further discussion could strengthen the mechanistic interpretation.

Response: We have expanded the discussion as suggested (end of section "Conserved protein–ligand recognition mechanism of the MLH1–MIP-box motif"). The weak intrinsic MLH1–MSH3-MIP interaction may create a permissive regulatory window, allowing a higher-affinity competitor like FAN1-MIP to effectively modulate the MMR activity at specific loci like CAG repeats. Regarding S2 site mutation (e.g., E669A), we note a key interpretative challenge: this site is a common hub for multiple MMR proteins (EXO1, MSH3). Therefore, while such mutations impair MMR and repeat stability, the phenotype reflects the loss of several interactions. Disentangling the specific role of FAN1 sequestration will require future, more targeted cellular models.

This manuscript reports the crystal structures of the human MLH1 C-terminal domain (MLH1-CTD) in complex with either the FAN1 MIP or MIM peptide. MLH1, a central component of the mismatch repair pathway, also plays a key role in regulating short nucleotide repeat instability in humans. When MLH1 associates with MutSβ, it promotes repeat expansion; in contrast, when MLH1 engages the nuclease FAN1, the complex suppresses expansions and instead promotes repeat contractions. The authors have further validated the MLH1–FAN1 interacting interfaces through mutagenesis, isothermal titration calorimetry (ITC), bio-layer interferometry (BLI), and cellular co-immunoprecipitation assays. Using classical biochemical approaches combined with crystallography, the study convincingly delineates how MLH1 and FAN1 interact to mediate their function in limiting the expansion of short nucleotide repeats.

It would strengthen the manuscript to examine whether the newly identified s3 site in human MLH1 affects repeat stability in cellular models. Nevertheless, the identification of a previously uncharacterized MLH1 binding surface (site s3), together with the experimental evidence and clear presentation, supports publication in *Nature Communications.* However, explanation of some experiment design and revision to address the points outlined below would enhance the clarity and impact of the work.

1. **Mutation design of 3A and 4A in MLH1-CTD**
   The MLH1_3A and 4A mutations were designed to disrupt the MLH1–FAN1 (MIM) interface. When the 3A and 4A mutations are displayed on the 2.2 Å crystal structure of MLH1-CTD (PDB: 3RBN), one residue in each patch belongs to the hydrophobic core (L636A in MLH1_3A and L639A in MLH1_4A) (see left).

   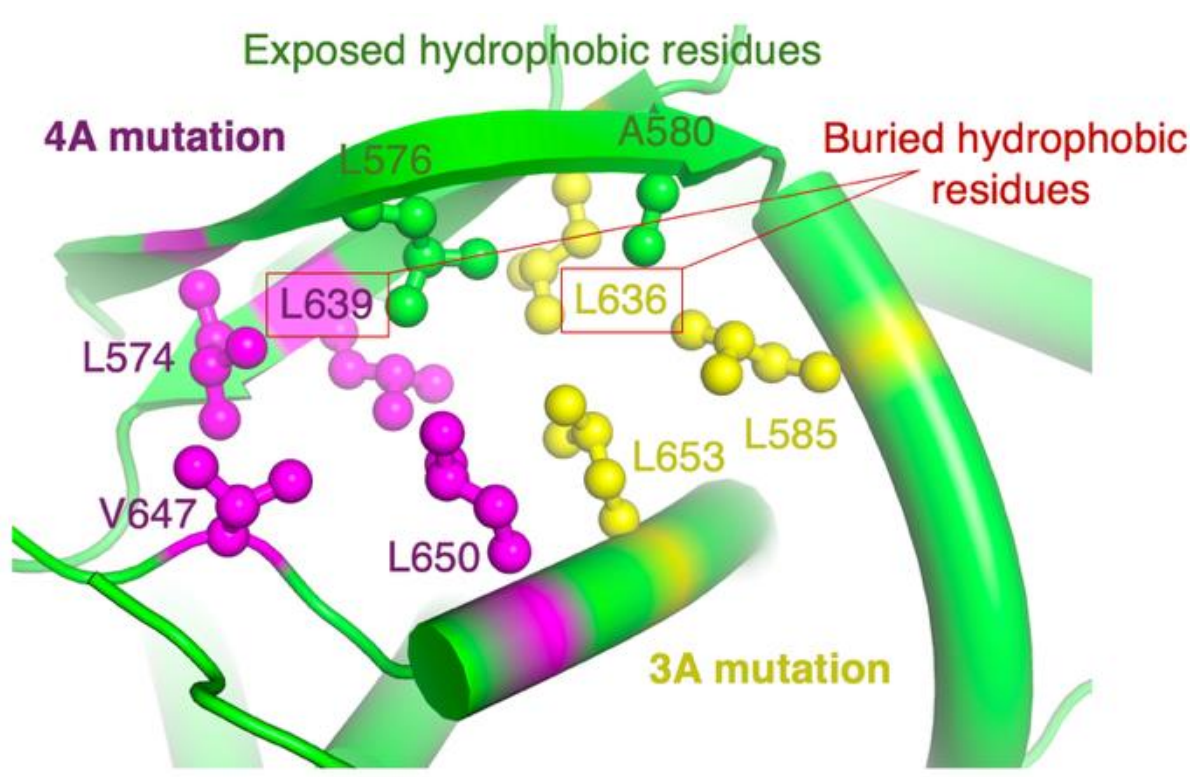

   Mutating them would destabilize the MLH1-CTD structure, which may explain the formation of inclusion bodies of these mutant proteins when over-expressed. The authors need to explain the nature of these mutations and the effects of a destabilized MLH1-CTD on its reduced binding to FAN1. Surprisingly, L576, which is exposed and covers L636 and L639, is not mutated to Ala or charged sidechain to verify the importance of site s3. A diagram showing the locations of the mutated residues in MLH1-3A and 4A and their solvent accessibility is needed.
2. **Potential influence of MutL dimerization on MLH1–FAN1 interactions**
   MLH1-CTD forms constitutive heterodimers with its partners PMS2, PMS1, or MLH3. The MutL dimerization interface typically extends beyond the s1 site and involves the extreme C-terminus. Could dimerization within MutL complexes influence, either positively or negatively, the interaction between human MLH1 and FAN1? Clarification or discussion of this possibility would be helpful.
3. **Placement and visualization of MLH1 binding-site summary**
   The combined summary of the three MLH1-CTD binding sites appears only in

Fig. 8e. Because these sites are central to the experimental design and conclusions, presenting this figure panel earlier in the manuscript would improve readability. The authors may consider illustrating their spatial relationships from more than one orientation, ideally with electrostatic surface potentials, to better convey the nature of the interactions.

4. **Inclusion of peptide electron densities**
   Given the moderate resolution of the crystal structures, including electron density maps for the MIP and MIM peptides in Fig. 2a and Fig. 4a would convince readers of the modeled interactions.
5. **Effects of FAN1 on mismatch repair**
   Interactions between MutSβ and MutL complexes are essential for mismatch repair. The manuscript reports substantially weaker binding of MLH1-CTD to the MSH3-MIP peptide compared to FAN1-MIP. What are the implications of such weak MLH1–MSH3 interactions? What functional consequences would be when the MLH1 s2 site (the MIP-binding region) is mutated with respect to mismatch repair efficiency and repeat stability? Further discussion could strengthen the mechanistic interpretation.
